# Fast and near-optimal monitoring for healthcare acquired infection outbreaks

**DOI:** 10.1371/journal.pcbi.1007284

**Published:** 2019-09-16

**Authors:** Bijaya Adhikari, Bryan Lewis, Anil Vullikanti, José Mauricio Jiménez, B. Aditya Prakash

**Affiliations:** 1 Department of Computer Science, Virginia Tech, Blacksburg, Virginia, United States of America; 2 Biocomplexity Institute & Initiative, University of Virginia, Charlottesville, Virginia, United States of America; 3 Department of Computer Science, University of Virginia, Charlottesville, Virginia, United States of America; 4 Department of Systems Engineering, United States Military Academy, West Point, New York, United States of America; Northeastern University, UNITED STATES

## Abstract

According to the Centers for Disease Control and Prevention (CDC), one in twenty five hospital patients are infected with at least one healthcare acquired infection (HAI) on any given day. Early detection of possible HAI outbreaks help practitioners implement countermeasures before the infection spreads extensively. Here, we develop an efficient data and model driven method to detect outbreaks with high accuracy. We leverage mechanistic modeling of *C. difficile* infection, a major HAI disease, to simulate its spread in a hospital wing and design efficient near-optimal algorithms to select people and locations to monitor using an optimization formulation. Results show that our strategy detects up to 95% of “future” *C. difficile* outbreaks. We design our method by incorporating specific hospital practices (like swabbing for infections) as well. As a result, our method outperforms state-of-the-art algorithms for outbreak detection. Finally, a qualitative study of our result shows that the people and locations we select to monitor as sensors are intuitive and meaningful.

## Author summary

Healthcare acquired infections (HAIs) lead to significant losses of lives and result in heavy economic burden on healthcare providers worldwide. Timely detection of HAI outbreaks will have a significant impact on the health infrastructure. Here, we propose an efficient and effective approach to detect HAI outbreaks by strategically monitoring selected people and locations (sensors). Our approach leverages outbreak data generated by calibrated mechanistic simulation of *C. difficile* spread in a hospital wing and a careful computational formulation to determine the people and locations to monitor. Results show that our approach is effective in detecting outbreaks.

## Introduction

Since the time of Hippocrates, the “father of western medicine”, a central tenet of medical care has been to “do no harm.” Unfortunately, the scourge of healthcare acquired infections (HAI) challenges the medical system to honor this tenet. When patients are hospitalized they are seeking care and healing, however, they are simultaneously being exposed to risky infections from others in the hospital, and in their weakened state are much more susceptible to these infections than they would be normally. Acquiring these infections increases the chances of either dying or becoming even sicker, which also lengthens the time the patient needs to stay in the hospital (increasing costs). These infections can range from pneumonia and gastro-intestinal infections like *Clostridium difficile* to surgical site infections and catheter associated infections, which puts nearly any patient in the hospital at risk. Antibiotic treatments intended to aid in recovery from one infection, may open the door for increased risk of infection from another.

Healthcare acquired infections are a significant problem in the United States and around the world. Some estimates put the annual cost between 28 and 45 billion US dollars per year in the US [1]. More importantly, they inflict a significant burden on human health. A recent study estimated more than 2.5 million new cases per year in Europe alone, inflicting a loss of just over 500 disability-adjusted life years (DALYS) per 100,000 population [2]. Given their burden and cost, their prevention is a high priority for infection control specialists. A simple approach to monitor HAI outbreaks would be to test every patient and staff in the hospital and swab every possible location for HAI infection. However, such a naive process is too expensive to implement. A better strategy is required to efficiently monitor HAI outbreaks.

A recent review article [3] included 29 hospital outbreak detection algorithms described in the literature. They found these fall into five main categories: simple thresholds, statistical process control, scan statistics, traditional statistical models, and data mining methods. Comparing the performance of these methods is challenging given the myriad diseases, definitions of outbreaks, study environments, and ultimately the purpose of the studies themselves. However, the authors identify that few of these studies were able to leverage important covariates in their detection algorithms. For example, including the culture site or antibiotic resistance was shown to boost detectability. Past simulation based approaches [4] tackle optimal surveillance system design, by choosing clinics as sensors, to increase sensitivity and time to detection for outbreaks in a population. In contrast, our approach selects most vulnerable people and locations to infections as sensors to detect outbreaks in a hospital setting. Different kinds of mechanistic models have also been used for studying HAI spread [5, 6, 7, 8]. Most of these are differential equation based models. We refer to [9] for a review of mechanistic models of HAI transmission.

On a broader level, sensor selection problem for propagation (of contents, disease, rumors and so on) over networks has gained much attention in the data mining community. Traditional sensor selection approaches [10, 11] typically select a set of nodes which require constant monitoring. Instead, in this paper, we select sensor set as well as the rate to monitor each sensor. Hence, our approach is novel from the data mining perspective as well. Recently Shao et al. [12] proposed selecting a set of users on social media to detect outbreaks in the general population. Similarly, Reis et al. [13] proposed an epidemiological network modeling approach for respiratory and gastrointestinal disease outbreaks. Other closely related data mining problems include selecting nodes for inhibiting epidemic outbreaks (vaccination) [14, 15, 16] and inferring missing infections in an epidemic outbreak [17].

We employ a simulation and data optimization based approach to design our algorithm and to provide robust bounds on its performance. Additionally, our simulation model is richly detailed in terms of the class of individuals and locations where sampling can occur. None of the prior works explicitly model the multiple pathways of infections for HAI outbreaks and fail in separating the location contamination and infections in people. We formalize the sensor set problem as an optimization problem over the space of rate vectors, which represent the rates at which to monitor each location and person. We consider two objectives, namely the probability of detection and the detection time, and show that the prior satisfies a mathematical property called submodularity, which enables efficient algorithms. In addition, we leverage data generated from a carefully calibrated simulation using real data collected from a local hospital. Our extensive experiments show that our approach outperforms the state-of-the-art general outbreak detection algorithm. We also show that our approach achieves the minimum outbreak detection time compared to other alternatives. To the best of our knowledge, we are the first to provide a principled data-driven optimization based approach for HAI outbreak detection. Though we validate our approach for a specific HAI, namely *C. difficile*, our general approach is applicable for other HAIs with similar disease model as well.

## Materials and methods

### Data

**Fig 1:**
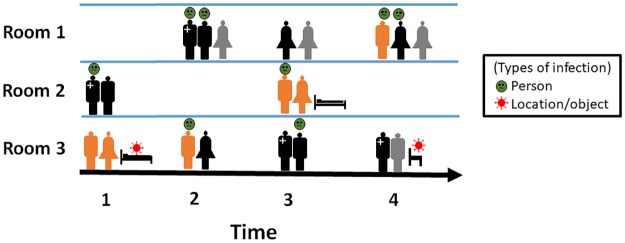
Visualization of a possible HAI spread. The only healthcare worker in the example is marked with a ‘+’ sign. The rest of the humans are patients and visitors. Each individual is assigned a unique combination of color and silhouette pair. Infected humans are indicated by the green emoji and contaminated locations are indicated by the bacteria emoji. As human agents move through various locations, they infect other agents and contaminate the locations.

As previously mentioned, we propose a data-driven approach in selecting the sensors. There are multiple challenges in obtaining actual HAI spread data such as high cost, data sparsity, and the ability to safeguard patient personal information. For this reason, we rely on simulated HAI contagion data. We use a highly-detailed agent-based simulation that employs a mobility log obtained from local hospitals [18, 19] to produce realistic contagion data. All the steps of this methodology are described in detail in [18], and we summarize them below for completeness. Fig 1 shows a visualization of simulated HAI spread. In this simulation, people (human agents) move across various locations (static agents) as defined by the mobility log and spread HAI in stochastic manner.

The simulation was developed in the following three steps: design of an *in-silico* or computer-based population and its activities, conceptualization of a disease model for a pathogen of interest, and the employment of a highly-detailed simulation. The following sections describe the data creation process in more detail.

#### *In-silico* population and mobility log

The first step in developing an HAI contagion model is the creation of a complete hospital population based on realistic hospital parameters. This will ensure high resolution and fidelity for the simulation. The hospital population was initially developed by capturing actual patient schedule data from regional hospitals in Southwest Virginia. The de-identified data contains information regarding patient location, disposition, and activities within a hospital. In addition to the patient schedule, the population incorporates the activities of healthcare workers such as nurses, doctors, therapists, and environmental services personnel among others. Their activities were compiled into the population based on direct observation of each type of healthcare worker. One additional level of realism was added to the population by including detailed information of locations, such as patient rooms and static objects such as furniture. We also use the term *fomite* to mean a location. The locations/fomites were modeled as static agents.

The final mobility log of the hospital population consists of a detailed database that specifies the type of agent, its location, specific agent, and the duration of the activity. Formally, the mobility logs are represented as a bipartite temporal network *G*(*P*, *L*, *E*, *T*), with partition *P* representing population of human agents, partition *L* representing locations, *E* representing who-visits-what-location relationship and *T* representing time/duration of the visit. The summary statistics of the mobility log and the resulting social networks as as follows:

As shown in Table 1, the mobility log includes 72,146 unique locations with 96,281 unique agents, and their interaction for the total duration of 200 days. The high spread in the average number of visits per location is due to the fact that some locations for example reception and staff room tend to get more visitors than others such as patients room. From the mobility log 200 social networks were created, one for each day. In these social networks, two agents have an edge between them if they were in the same location at the same time. The summary statistics of these social networks are presented in Table 1 as well.

**Table 1:** Summary statistics on the mobility log and the resulting social networks.

total no. of locations	72,146
total no. of agents	96,281
total no. of days	200
average no. of mobility entries per day	138,765.4 ± 6384.15
average no. of visits per location	384.6 ± 1949.22
average no. of nodes in social networks	6,924.8 ± 96.51
average no. of edges in social networks	45,503.8 ± 4267.97
average degree in social networks	13.2 ± 1.44
average clustering co-efficients in social networks	0.4 ±0.03

#### Disease model

**Fig 2:**
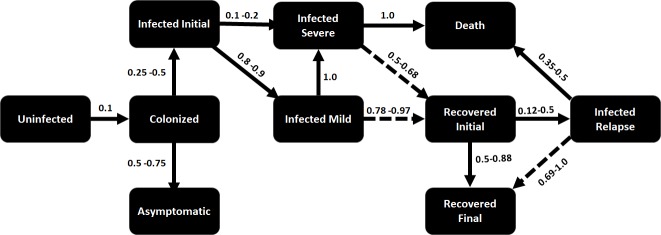
Human infection model for *C. difficile*. Each state in the finite state machine shown above indicates the stages in the infection/recovery process. The arrows indicate possible transition in state and the weight on the arrows indicate the transition probability. The dashed arrows represent transition under medication.

The next step in generating a realistic simulation is to model the disease accurately. Mathematical models that have been proposed for the spread of *C. difficile* in a hospital are either differential equation-based, e.g., [5], or agent-based, e.g., [19, 20, 21, 22]. We use an agent-based approach, in which the disease model is a probabilistic finite-state machine (FSM). The disease model was developed based on recent studies on *C. difficile* infections [23, 24, 25, 26, 27]. Different health states (shown in Fig 2) and various transition probabilities were taken from these studies. Once the simulation is initiated, each agent will move through the different disease/health states described in the disease model beginning with the uninfected state. As the simulation progresses, agents are either colonized or not with the *C. difficile* bacterium. It is important to note that the transition probabilities for each disease/health state utilize current infection and recovery rates to capture the actual behavior of the pathogen in the hospital setting. Fig 2 shows the disease model for humans.

**Fig 3:**
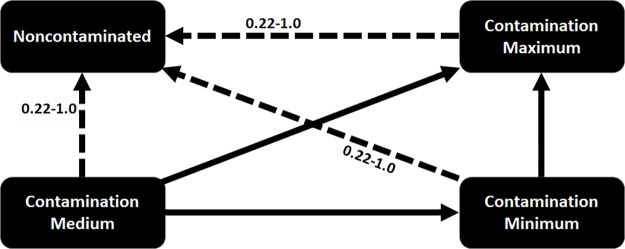
Fomite contamination model for *C. difficile*. Each state in the finite state machine shown above indicates the stages in the contamination/decay process. The solid arrows indicate possible transition in state. The transition between the states depend on number of infected people in the location. The dashed arrows represent transition assuming cleaning.

An important characteristic of this model is that agents consist of both people and location/objects (e.g., fomites), and there are two pathways for the disease to spread, namely, person to person, and person to location. While differential equation-based models have typically only represented person to person transmission, recent agent-based models have considered these two pathways, e.g., [21, 22]—they represent rooms in the hospital, along with the dynamics of pathogen load in these rooms. Since the infection in people and the contamination of fomites are inherently different [21, 22], we use two separate FSMs to model the spread of the pathogen throughout the hospital. The fomite FSM includes states capturing low, mid, and high level of contamination, which can trigger infection on human agents in the simulation. Fig 3, shows the disease model for fomites in different hospital locations. These carefully designed FSMs with meticulously calibrated transition probabilities along with the agent mobility logs constitute the input for the simulation software to generate the HAI contagion.

#### Simulation

The final step in the simulation process is to use a powerful simulation software capable of processing a large amount of agents through the disease models. This study utilizes EpiSimdemics [28], which is a high performance computing based simulation software. EpiSimdemics is an agent based simulation, in which every person is represented as an agent. Each agent performs activities as described by a master schedule. The schedule determines the location of each agents at a given time, at the resolution of seconds. If two or more agents are in the same location, then they can interact with each other. For example, an infected agent can infect a susceptible agent if they are in the same location based on the transition probabilities of the disease/health state. EpiSimdemics and its extensions have been used in wide range of studies from informing pandemic influenza policy [29], to modeling the immune system in the gut [30], to assisting in the design of surveillance systems [4]. This platform has been shown to scale to what was a record-breaking level [31] for an epidemic model.

We briefly mention details of model calibration and validation, following the description in [18]. The model is calibrated to capture the correct dynamics of *C. difficile* outbreak; community-acquired and hospital-acquired cases were collected to calibrate the simulation parameters. It was ensured that the simulation output of hospital-acquired cases closely matched the real value, given the community-acquired cases. The model is validated at various levels. First, very detailed data on mobility and activities of patient, healthcare workers, and visitors is used. These, and fomite behaviors, were estimated through direct observations at the hospital. Finally, feedback from the Hospital Infection Preventionist and healthcare workers was used to ensure that the dynamics of the simulation behaved in a similar manner to the actual movements of patients and healthcare workers during normal hospital operations.

#### Simulation outputs

We ran EpiSimdemics multiple times with various initial conditions. Each simulation instance produced a cascade (infection dendogram) consisting of information on identity of newly infected agents, time of infection, the source of infection, and the activity the agent was performing when infected. In the following, we use the terms *simulation instances* and *cascades* interchangeably. Each simulation also produces information regarding the infection status of each agent on each day for all 200 days. Formally, we have a set I of individual simulation instances *i* of HAI spread. Each instance *i* is a simulation of HAI spread starting from a particular initial stage. Overall, the average number of infection per simulation is 22.94, with a median of 23.32 and a standard deviation of 6.92.

**Fig 4:**
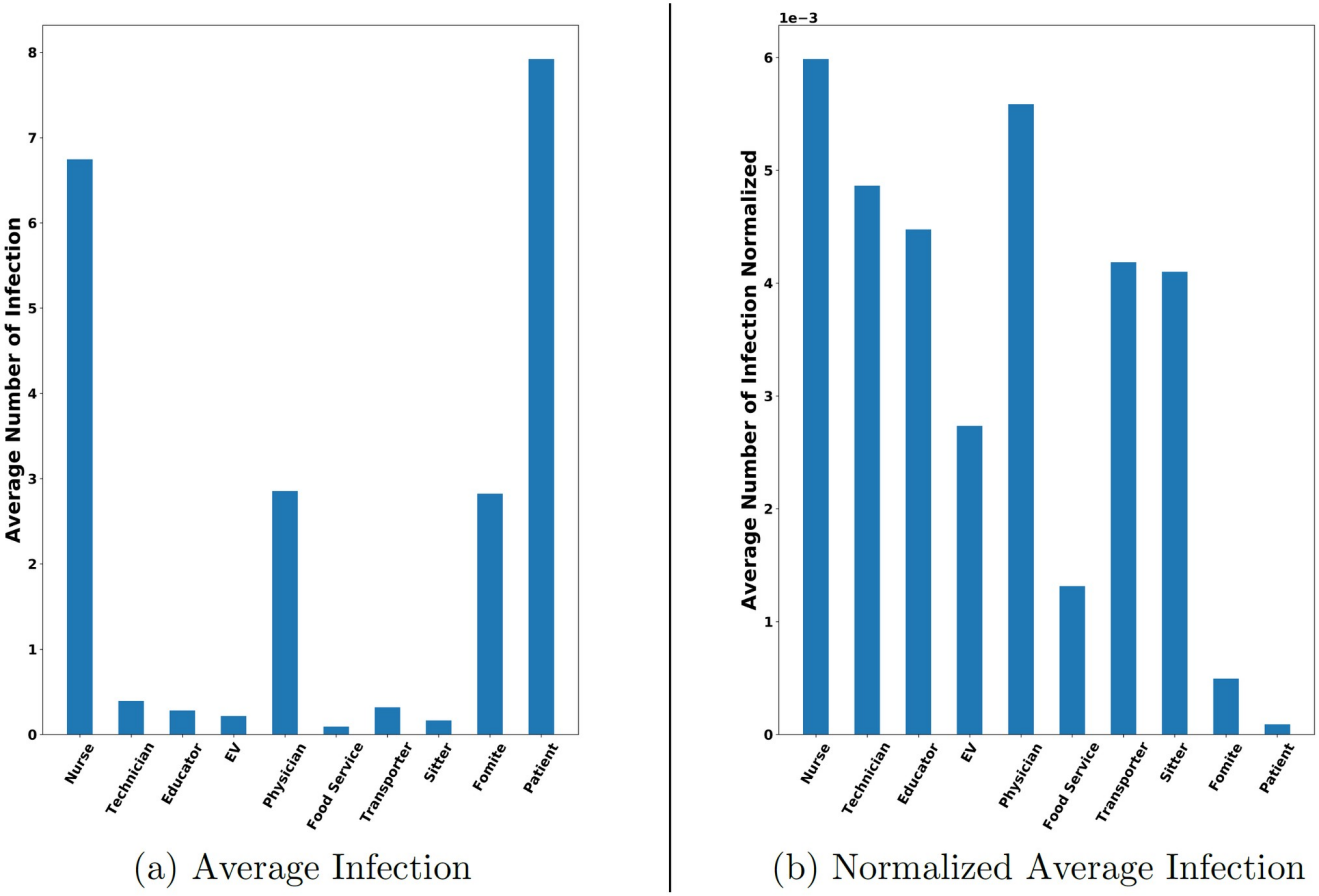
Infection distribution for various categories of agents. Note that “EV” stands for external visitors. (a) Infection for each agent category averaged across simulation instances. (b) Average infection normalized by the number of agents in each category. High susceptibility of healthcare workers can be attributed to their high mobility. Patients have the highest number of infections on average despite having a low normalized infection rate as they are the largest population group.

Fig 4 shows the distribution of infections over different categories of agents. The infections are dominated by nurses, physicians, fomites, and patients. Patients have the highest infection rate as they are the largest group by population. Nurses and physicians have a high infection levels as well. This is because they are more mobile and hence are more exposed to HAIs. They also have higher interactions with other health care workers.

In summary, our HAI contagion simulation data follows meticulous stages of development, modeling and calibration. The simulation results produce intuitive and realistic infection patterns. While this case of *C. difficile* in a particular hospital in Virginia is quite specific, these results are generalizable. While the particulars of movement in different hospitals and dynamics of different diseases may alter the specific values of our findings the general trends will be similar. The infection control practices and rhythms of patient care in an intensive care unit are similar across hospitals, and at the moment *C. difficile* remains a major contributor to healthcare acquired infections.

### Sensor set and rate selection

Recall that our goal is to select a set of agents as sensors, and *the rate at which each such sensor should be monitored*, such that future HAI outbreaks are detected with high probability, and as early as possible. However, these have to be selected within given resource constraints. We start with a formalization of these problems. Finding a minimum cost sensor set is a challenging optimization problem, and we present efficient algorithms by using the notion of submodularity.

We first define some notations. Let bold letters represent vectors. Let *P* and *L* denote the sets of human agents and locations respectively; let *n* = |*P* ∪ *L*|—this will be the total number of agents in our simulations. Let *B* denote the budget on a number of samples that is permitted (weighted by cost of agents), i.e, it is the sum of expected number of swabs to detect whether a location is contaminated or a human is infected. As mentioned earlier, the mobility logs are represented as a bipartite temporal network *G*(*P*, *L*, *E*, *T*), with two partitions *P* and *L* representing agents, *E* representing who-visits-what-location relationship and *T* representing time/duration of the visit. We consider each agent to be a *node* in the temporal network *G*. Hence we use the terms *node* and *agent* interchangeably. Now, let **c** ∈ *R*^*n*^, be the vector of costs, i.e., **c**[*v*] is the cost of monitoring node *v*. Let **r** ∈ *R*^*n*^ be the vector of monitoring rates, where **r**[*v*] denotes that the probability that node *v* is monitored (e.g., swabbed) each day. Finally, let *T*_*max*_ denote the maximum time in each simulation instance.

#### Optimizing probability of detection

Consider an arbitrary simulation instance *i*, an arbitrary sensor set and associated monitoring rate vector **r**. Agent *v* is monitored at the rate of **r**[*v*]. Let the number of days in which node *v* is in the infected state in simulation instance *i* be *τ*(*v*, *i*). Then, the probability that node *v* is detected in simulation *i* is

$$\begin{array}{c} P_{d}\left(v|i,r\right)=1-{\left(1-r\left[v\right]\right)}^{\tau (v,i)} \end{array}$$ (1)

Next, the probability of at least one node being detected in simulation *i*, given the rate vector **r**, is

$$P(i|r)=1-\prod_{v\in P\cup L}\left(1-P_{d}\left(v|i,r\right)\right)$$ (2)

$$P(i|r)=1-\prod_{v\in P\cup L}[1-(1-{\left(1-r\left[v\right]\right)}^{\tau (v,i)})]$$ (3)

$$P(i|r)=1-\prod_{v\in P\cup L}{\left(1-r\left[v\right]\right)}^{\tau (v,i)}$$ (4)

Recall that I denotes the set of simulations. It follows that the expected number of cascades, where at least one node is detected, given the rate vector **r**, is $\sum_{i\in I}$ *P*(*i*|**r**). The probability that a sensor node is detected equals the fraction of cascades in which at least one sensor node is infected. This motivates the first problem we study.

**Problem 1**. *Find the vector* r*, *such that*

$$\begin{array}{c} r^{*}=\underset{r}{\text{arg}\;\text{max}}\sum_{i\in I}P\left(i|r\right) \end{array}$$ (5)

*and* ∑_*v*_ **r**[*v*] ⋅ **c**[*v*] ≤ *B*.

#### Optimizing expected time of detection

As discussed above, the node *v* is sampled/swabbed for infection each day with rate **r**[*v*]. Let the ordered list of days in which node *v* is infected in simulation instance *i* be $\gamma \left(v,i\right)=\left\{t_{1}^{v},t_{2}^{v},\ldots t_{n}^{v}\right\}$, where $t_{i}^{v}$ represents the *i*^*th*^ day in which node *v* is infected.

Here, we are interested in the first day until node *v* is detected to be infected. On the days where node *v* is not infected, sampling node *v* does not result in outbreak detection. The detection time is the first time at which *v* is infected and is sampled. Since node *v* is sampled each day with probability **r**[*v*], the first time in *γ*(*v*, *i*) in which it gets detected (restricted to the times in which *v* is infected) is a geometric process. Therefore, in expectation, node *v* is detected to be infected on the 1/**r**[*v*]^th^ day in *γ*(*v*, *i*). Let that be denoted as *Det*(*v*|*i*, **r**). For example, let us consider a case when a node gets infected in day 5 of simulation and remains infected until day 15. During this time, if we sample this node with a rate of 0.2, the node is sampled and detected to be infected on the 5^*th*^ day of infection which is the day 9 of the simulation in expectation. If 1/**r**[*v*] is greater than the length of *γ*(*v*, *i*), we consider that the infection to be undetected and set *Det*(*v*|*i*, **r**) as *T*_*max*_, the last time-stamp in any of the simulation instances.

Formally, the minimum detection day for simulation instance *i* is,

$$\begin{array}{c} D\left(i|r\right)=\underset{v}{\text{min}}\;Det\left(v|i,r\right) \end{array}$$ (6)

Our goal is to minimize *D*(*i*|**r**) over all *i*, **r**. This turns out to be a challenging computational problem, and instead we consider its converse. Now, the problem to optimize for expected time of detection can be posed as following:

**Problem 2**. *Find the vector* r*, *such that*

$$\begin{array}{c} r^{*}=\underset{r}{\text{arg}\;\text{max}}\sum_{i\in I}[T_{max}-D\left(i|r\right)] \end{array}$$ (7)

*and* ∑_*v*_ **r**[*v*] ⋅ **c**[*v*] ≤ *B*.

### Our methods

Unfortunately, Problems 1 and 2 are both computationally very challenging. In fact, both the problems can be proven to be NP-hard.

**Lemma 1**. *Problem 1 is NP-hard*.

**Lemma 2**. *Problem 2 is NP-hard*.

We provide the proofs for both the lemmas in the supplementary, where we show that the NP-Complete SetCover problem can be viewed as a special case of both the Problems 1 and 2.

Since our problems are in the computational class NP-hard, they cannot be solved optimally in polynomial time even for simplistic instances, unless P = NP. The instances we need to consider are pretty large, so a naive exhaustive search for the optimal solution is also not feasible and will be too slow. Therefore, we focus on designing efficient near-optimal approximate solutions.

We begin with Problem 1. The function we are trying to optimize for problem 1 is defined over a discrete lattice, i.e. the rate vector **r**. Our approach is to show that this function is a submodular lattice function. The notion of submodularity, which is typically defined over set functions, can be extended to discrete lattice functions (e.g. recenty in [32]). Informally, submodularity means that the objective value has a property of diminishing returns for a small increase in the rate in any dimension. It is important to note that submodularity for lattice functions is more nuanced than for simple set functions (we define it formally in the Supplementary Information section). Fortunately, it turns out that this property implies that a natural greedy algorithm (which maximizes the objective marginally at each step) gurantees a (1 − 1/*e*)-approximation to the optimal solution. Without such a property, it is not clear how to solve Problem 1 efficiently even for a small budget.

We have the following lemma.

**Lemma 3**. *The objective in Problem 1 is a submodular lattice function*.

The detailed description of the submodularity property and proof of lemma 3 are presented in the supplementary.

Our HaiDetect algorithm for Problem 1 selects the sensors to be monitored and rates such that nodes which tend to get infected across multiple simulation instances have higher infection rates. Specifically, at each step, HaiDetect selects the node *v* and the rate *r* among all possible candidate pairs of nodes and rates, such that the average marginal gain is maximized. HaiDetect keeps adding nodes and/or increasing the rates to monitor the selected nodes until the weighted sum of the rates is equal to the budget *B*. The detailed pseudocode is presented in Algorithm 1.

**Algorithm 1** HaiDetect

**Require**: *I*, budget *B*

1: **for** each feasible initial vector **r**_0_ **do**

2:  Initialize the rate vector **r** = **r**_0_

3:  **while** ∑_*v*_ **r**[*v*] ⋅ **c**[*v*] < *B* **do**

4:   Find a node *v* and rate *r* maximizing average marginal gain

5:   Let **r**[*v*] = *r*

6:   Remove all candidate pairs of nodes and rates which are not feasible

7: Return the best rate vector **r**

HaiDetect has desirable properties in terms of both effectiveness and speed. The performance guarantee of HaiDetect is given by the following lemma.

**Lemma 4**. HaiDetect *gives a (1-1/e) approximation to the optimal solution*.

The lemma above gives an offline bound on the performance of HaiDetect, i.e., we can state that the (1-1/e) approximation holds even before the computation starts. We can actually obtain a tighter bound by computing an empirical online bound (once the solution is obtained) which can be derived using the submodularity and monotonicity of Problem 1. For us to state the empirical bound, let us define some notations.

Let the solution selected by HaiDetect for a budget *B* be $\hat{r}$. Similarly, let the optimal vector for the same budget be r*. For simplicity, let the objective function in Problem 1 be *R*(⋅). For all nodes *v* and for *a* ∈ [0, 1], let us define Δ_*v*_ as follows:

$$\begin{array}{c} \Delta_{v}=\underset{a}{\text{max}}\;[R\left(\hat{r}\vee a\cdot \chi^{\left\{v\right\}}\right)-R\left(\hat{r}\right)] \end{array}$$ (8)

Similarly let us define *σ*_*v*_ as the argument which maximizes Δ_*v*_

$$\begin{array}{c} \sigma_{v}=\underset{a}{\text{arg}\;\text{max}}\;[R\left(\hat{r}\vee a\cdot \chi^{\left\{v\right\}}\right)-R\left(\hat{r}\right)] \end{array}$$ (9)

Now, let $\delta_{v}=\frac{\Delta_{v}}{c\left[v\right]\cdot \sigma_{v}}$. Note that for each node *v*, there is a single *δ*. Let the sequence of nodes *s*_1_, *s*_2_, …, *s*_*n*_ be ordered in decreasing order of *δ*_*v*_. Now let *K* be the index such that $\theta =\sum_{i=1}^{K-1}c\left[s_{i}\right]\sigma_{s_{i}}\leq B$ and $\sum_{i=1}^{K}c\left[s_{i}\right]\sigma_{s_{i}}>B$. Now the following lemma can be stated.

**Lemma 5**. *The online bound on R*(r*) *in terms of the current rate* $\hat{r}$ *assigned by* HaiDetect *is as follows*:

$$\begin{array}{c} R\left(r^{*}\right)\leq R\left(\hat{r}\right)+\sum_{i=1}^{K-1}\Delta s_{i}+\frac{B-\theta }{c\left[s_{K}\right]\sigma_{s_{K}}}\Delta_{s_{K}} \end{array}$$

The lemma above allows us to compute how far the solution given by HaiDetect is from the optimal. We compute this bound and explore the results in detail in the Results section. In addition to the performance guarantee, HaiDetect’s running time complexity is as follows.

**Lemma 6**. *The running time complexity of* HaiDetect *is O*(*c* ⋅ *B*^2^(|*P*| + |*L*|)), *where c is the number of unique initial vectors* **r**_0_, *B is the budge, P is the set of human agents and L is the set of locations*.

Note that the constant *c* is much smaller than the total population, i.e., *c* << |*P*| + |*L*| in our case as infections are sparse and we do not need to consider agents and locations which never get infected. The most expensive computational step in Algorithm 1 is the estimation of the node *v* and rate *r* that gives the maximum average marginal gain (Step (i) of 1(b)). This can be expedited using lazy evaluations and memoization. Hence, the algorithm is also quite fast in practice. Moreover, it also embarrassingly parallelizable. The steps (a) and (b) for each initial vector can be performed in parallel.

We also propose a similar algorithm HaiEarlyDetect for Problem 2. The main idea here is that we assign higher rates to nodes which tend to get infected earlier in many simulation instances. The pseudocode for HaiEarlyDetect is presented in Algorithm 2.

**Algorithm 2** HaiEarlyDetect

**Require**: *I*, budget *B*

1: **for** each feasible initial vector **r**_0_ **do**

2:  Initialize the rate vector **r** = **r**_0_

3:  **while** ∑_*v*_ **r**[*v*] ⋅ **c**[*v*] < *B* **do**

4:   Find a node *v* and rate *r* minimizing the average detection time

5:   Let **r**[*v*] = *r*

6:   Remove all candidate pairs of nodes and rates which are not feasible

7: Return the best rate vector **r**

As shown in Algorithm 2, HaiEarlyDetect optimizes the marginal gain in the objective in Problem 2 in each iteration. It turns out that the objective in Problem 2 is not submodular. However, as shown by our empirical results, the greedy approach we propose works very well in practice and outperforms the baselines. Moreover, it too runs fast in practice as the same optimization techniques discussed earlier for HaiDetect applies to HaiEarlyDetect as well.

#### Baseline methods

We compare the performance of our approaches with data driven and natural baselines from practice.

#### Baseline from practice

We compare the performance of monitoring the sensors selected by HaiDetect and HaiEarlyDetect with different natural baselines motivated from practice. An obvious heuristic to monitor HAI outbreaks would be to monitor all the agents (human and fomites) every day. Other natural baselines we compare against include, monitoring all patients, swabbing all locations for fomites, monitoring all nurses, and so on. These methods get too expensive as the number of personnel and locations increase.

#### Data-driven baseline

An interesting baseline would be methods for general sensor set selection problems. Here we compare our approach against Celf [10], a state-of-the-art method for general sensor set selection problem. Celf was published in SIGKDD, a premiere data mining venue in 2007 and is still used to solve sensor set selection problem. We run Celf in the same set simulation as our methods. Celf is also a greedy algorithm designed for a submodular set function (as opposed to a lattice function in our case). Celf tries to add node *v* to the sensor set with rate **r**[*v*] = 1, such that the number of newly detectable simulation instances is maximized. CELF has been previously used for selecting sensors in water distribution network and in other network settings.

## Results

We ran HaiDetect and HaiEarlyDetect and compared them with the baselines in various settings for both qualitative and quantitative studies. We ask questions like: “How does the performance of the methods change with different budget constraints and with more data?”, “Are the sensors selected by our methods qualitatively the same?”, “Is there an advantage in using HaiDetect or HaiEarlyDetect over Celf?”, and so on.

### Quality of sensors w.r.t the bounds

**Fig 5:**
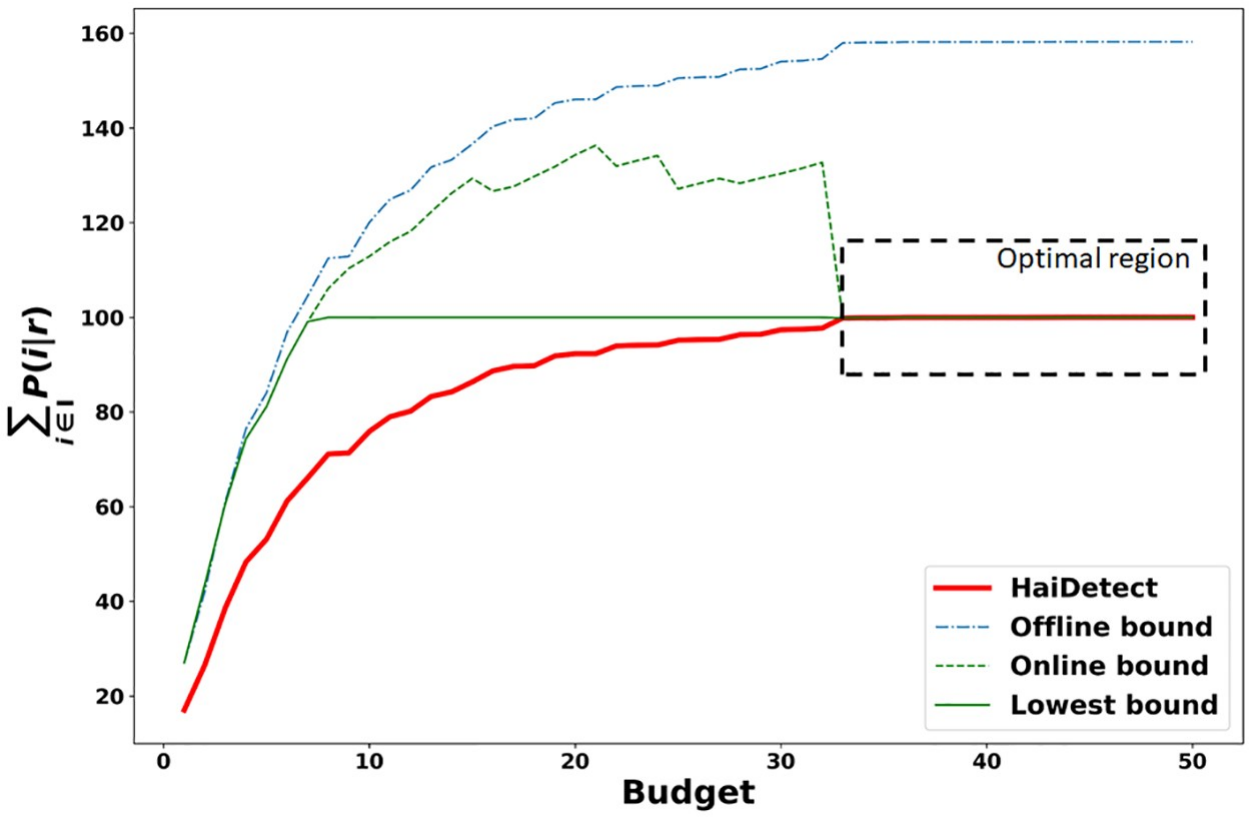
The objective value due to the solution returned by HaiDetect and the bounds for various budgets. The region in the dashed box shows where HaiDetect is optimal.

In the previous section, we discussed two types of bounds on the performance of HaiDetect. Here we show how far the solution given by HaiDetect is from the optimal value for various budgets. For this experiment, we ran HaiDetect on a set of 100 simulations and computed the value of the objective in Problem 1 for the resulting rate vector. We also computed the overall bound, based on (1 − 1/*e*) approximation and the empirical bound as per Lemma 5. Since the objective value cannot exceed the number of simulations, we also compute the lowest bound as the minimum of two bounds and the number of simulations. We repeat the experiment for budget size from 1 to 50. The resulting plot is presented in Fig 5.

Fig 5 highlights several interesting aspects. First of all, we can see that the online bound is always tighter than the offline bound. Moreover, we also observe that as the performance of HaiDetect reaches close to the optimal (with increase in budget), the online bound becomes more and more tight until both the performance and bound are equal, indicating the values of the budget for which HaiDetect solves Problem 1 optimally. This results demonstrates that HaiDetect can accurately find sensors which can detect any observed outbreaks given sufficient budget.

### Sensor quality with budget

Given that HaiDetect is near-optimal for the observed outbreaks, we evaluate its effectiveness in detecting unseen (“future”) outbreaks. Here, we compare the performance of HaiDetect and Celf with respect to the budget on “unobserved” simulations. For this experiment, we performed a 5-fold cross validation on 200 simulations. Specifically, we divided the simulations into 5 groups, and at each turn we selected the sensors in the first four groups and computed the sum of outbreak detection probability as shown in Eq 1 in the fifth group (the test set). Then we normalize the resulting sum of outbreak detection probability by total number of simulation instances in the the same group. The normalized value can be intuitively described as the average probability of detecting a future outbreak. We repeat this process five times ensuring each group is used for success evaluation. We then compute the overall average and its standard error. We repeat the entire process for the budgets from 1 to 50.

**Fig 6:**
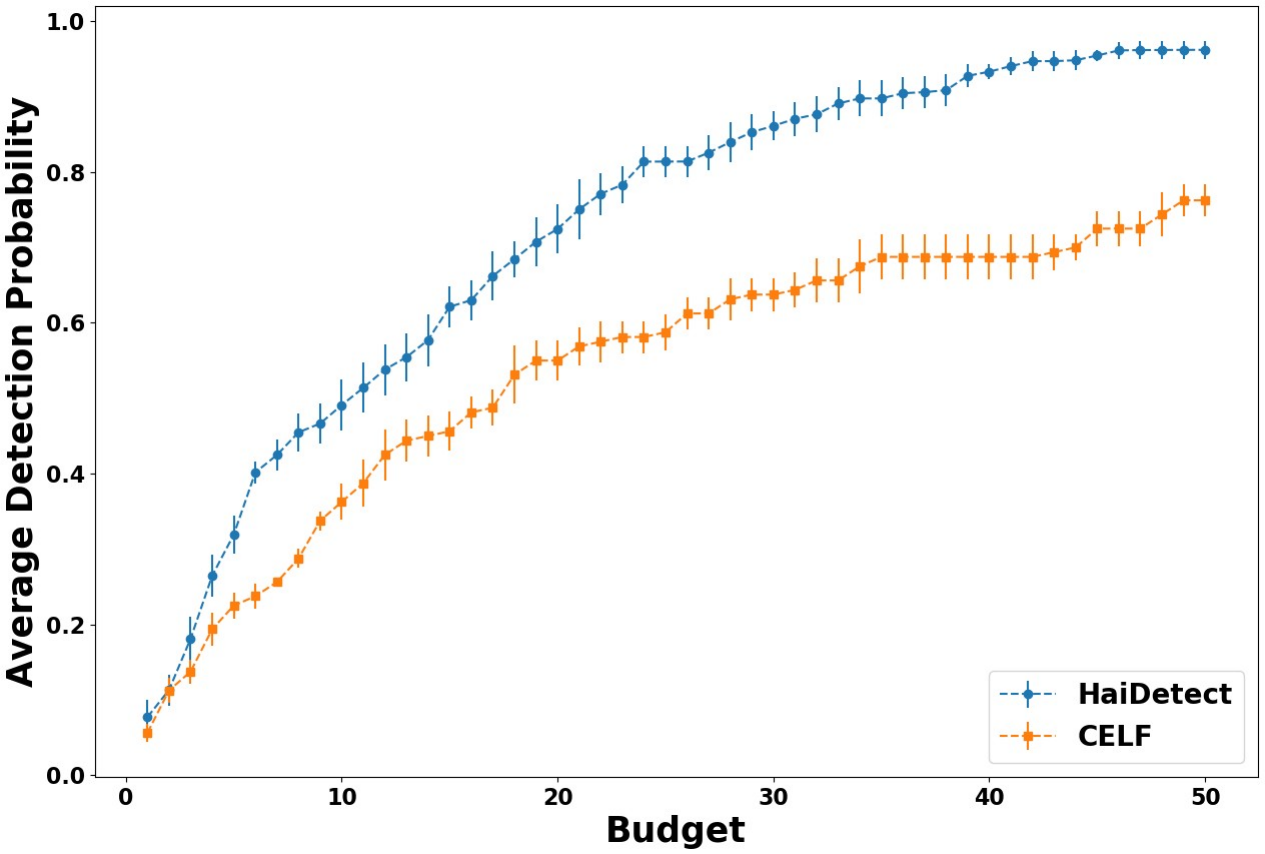
Probability of detecting future outbreaks (normalized) for different budgets. HaiDetect significantly outperforms Celf implying that monitoring sensors selected by HaiDetect have higher chance of detecting an outbreak.

The result of our experiment is show in Fig 6. The first observation is that HaiDetect consistently outperforms Celf for all values of the budget. The disparity between the methods is more apparent for larger values of budget. The difference in quality of the sensors can be explained by the fact that Celf only assigns rate of 0 or 1. However, HaiDetect can strategically assign non-integer rates so as to maximize the likelihood of detection.

We can also observe that the standard error for the HaiDetect decreases and is negligible for larger budgets. However, it is not the case for Celf. This shows that not only the quality of sensors detected by HaiDetect is better, but it is more stable as well. Finally, we see that probability of an outbreak being detected by sensors selected by HaiDetect is 0.96 when budget is equal to 50, whereas it is only around 0.75 for Celf. Similarly, a budget of only 25 is required to detect an outbreak with probability of 0.8 for HaiDetect. For the same budget, sensors selected by Celf detect cascades with probability of 0.55. The result highlights that HaiDetect produces more reliable monitoring strategy for HAI outbreak detection.

### Sensor quality with increasing simulations

Here, we investigate the change in performance of HaiDetect and Celf as the number of simulations used to detect the sensor increases. For this experiment, we used 150 distinct simulations. We divided the simulations into two categories, training and testing sets. We used the cascades in the training set to select the sensors and used the ones in the testing set to measure quality. First we decided on a budget of 10 and training size of 10 cascades. We ran both HaiDetect and Celf for this setting and measured the quality using the cascades in the testing set. We then increased the training size by 10 till we reached the size of 100. We repeated the same procedure for budgets of 30 and 50. We compute the average probability of detection in the same manner as described above.

**Fig 7:**
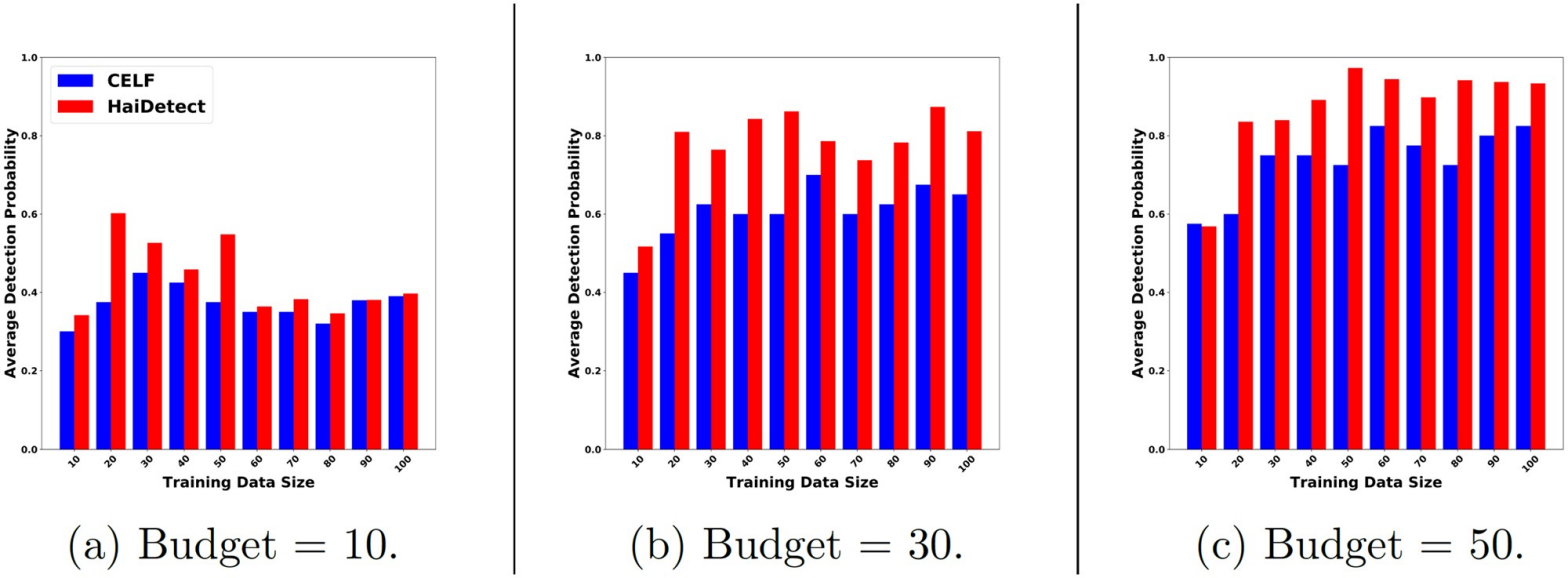
Average probability of detecting future outbreaks for sensors computed using T simulations (training) for different values of T, and tested on the remaining simulations for a budgets of (a) 10, (b) 30, and (c) 50. Note that HaiDetect required only 20 simulation instances to detect future outbreak with probability of 0.8.

Fig 7 summarizes the result. We can observe that HaiDetect outperforms Celf consistently. It reinforces the previous observation that HaiDetect selects good sensors for the HAI outbreak detection. An interesting observation is that the performance tails off after training size of 20 for larger budgets, which implies that not many cascades have to be observed before we can select good quality sensors. This is an encouraging finding as gathering large number of real cascades of HAI spread is not feasible.

**Fig 8:**
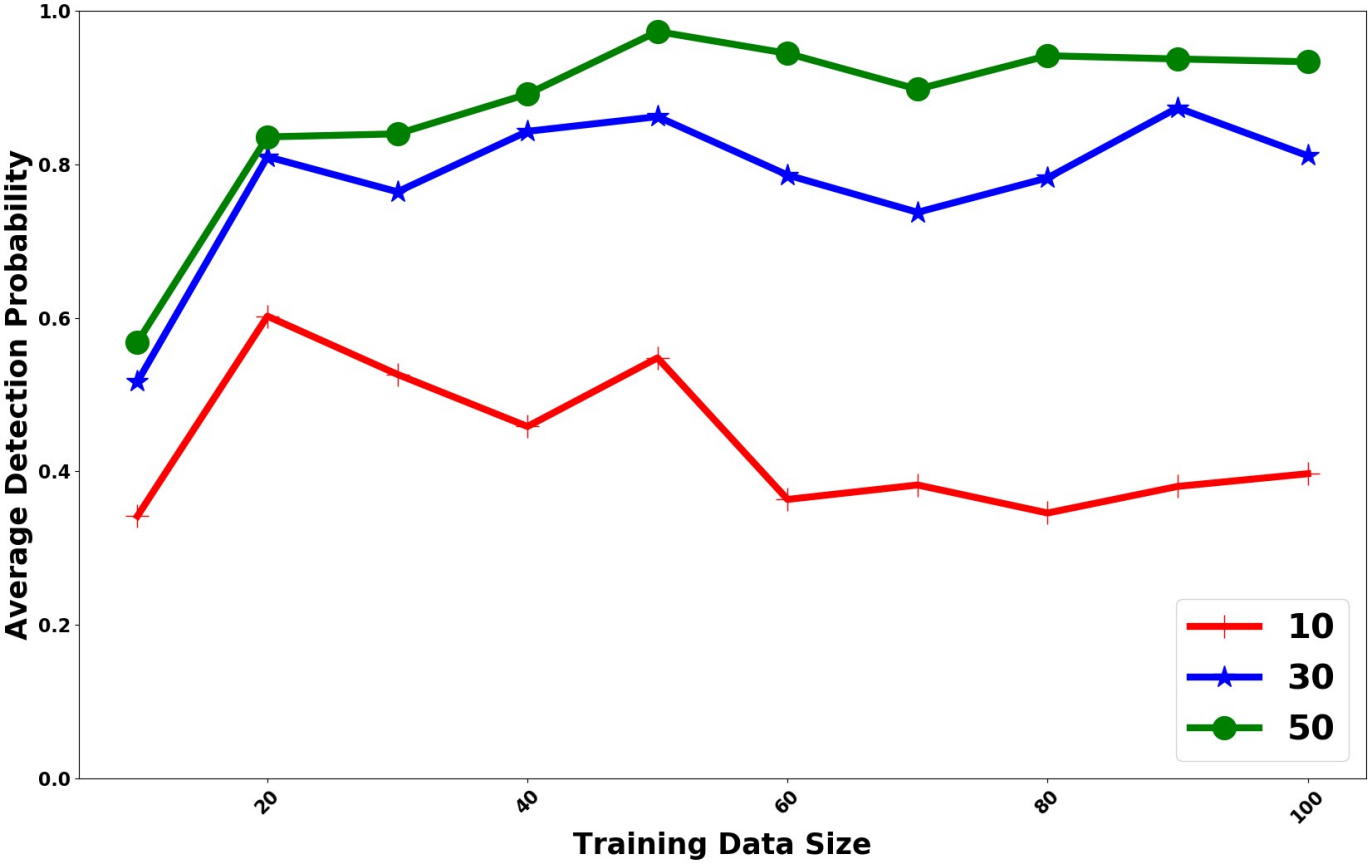
Average probability of detecting future outbreaks for sensor sets computed using T simulations (training), and evaluated using the remaining simulations for different budget values.

Next we study the change in performance of HaiDetect with the training size for various budget sizes. Here we tracked the performance of HaiDetect for budgets of 10, 30, and 50 for training sets of various size. The result is summarized in Fig 8.

As shown in the figure, the difference between peformance of HaiDetect for budgets 30 and 10 is much larger than that for budgets 50 and 30. The normalized objective, or the probability of detection, is close to 1 at budget 50, indicating that monitoring sensors at rates assigned by HaiDetect detects almost all the HAI outbreaks. Hence, in expectation, roughly 50 swabs a day is enough to monitor an outbreak in a hospital wing. Again, we observe that performance of HaiDetect tails off after the training size of 20. It provides extra validation for the observation that a limited number of observed cascades are enough to select high quality sensors.

### Time of detection

**Fig 9:**
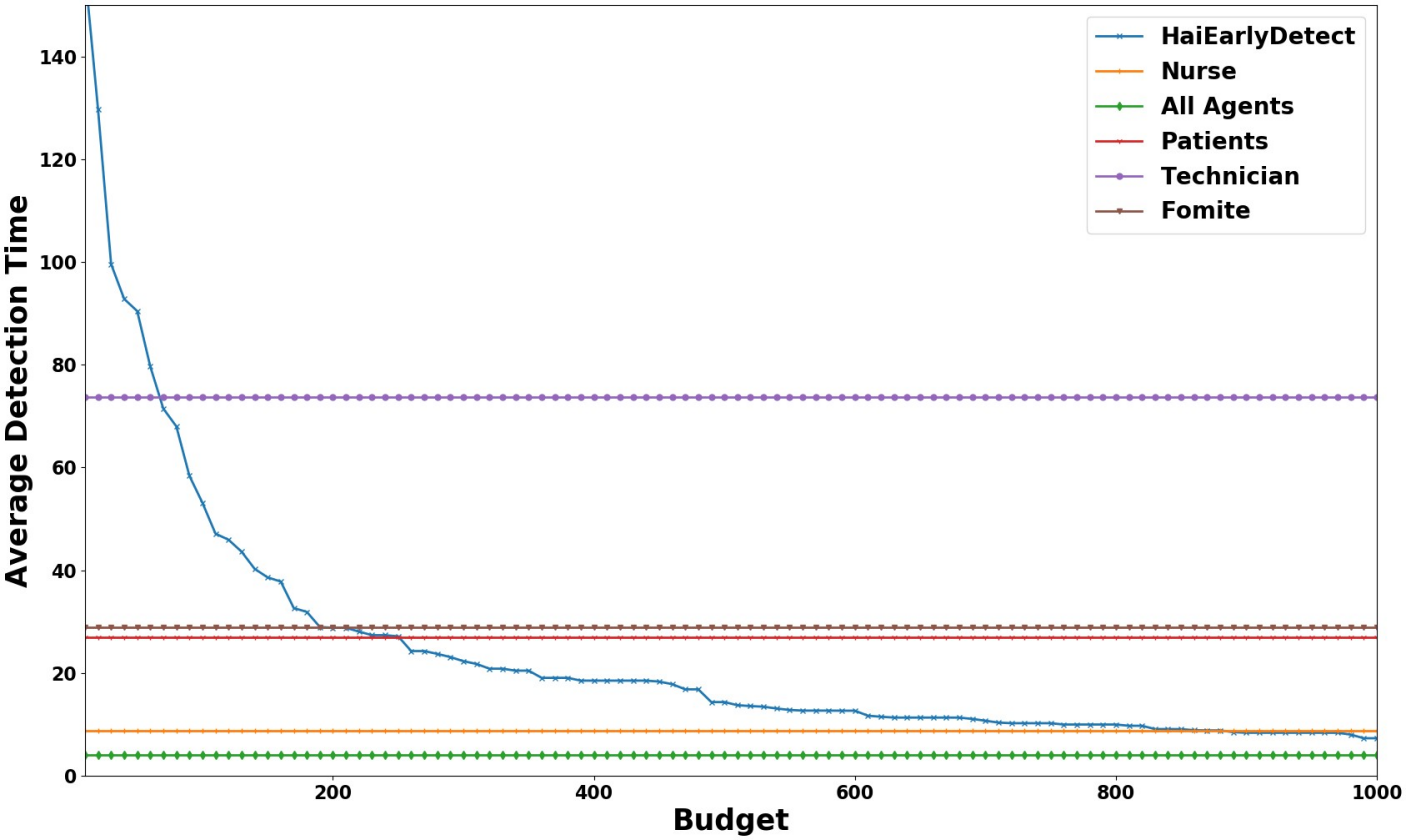
Average dectection time (in days) for various budgets. The flat lines are the detection time for monitoring all members of different categories of agents. Note that for a budget of 1000, monitoring sensors selected by HaiEarlyDetect detect future outbreaks earlier than monitoring all nurses.

**Fig 10:**
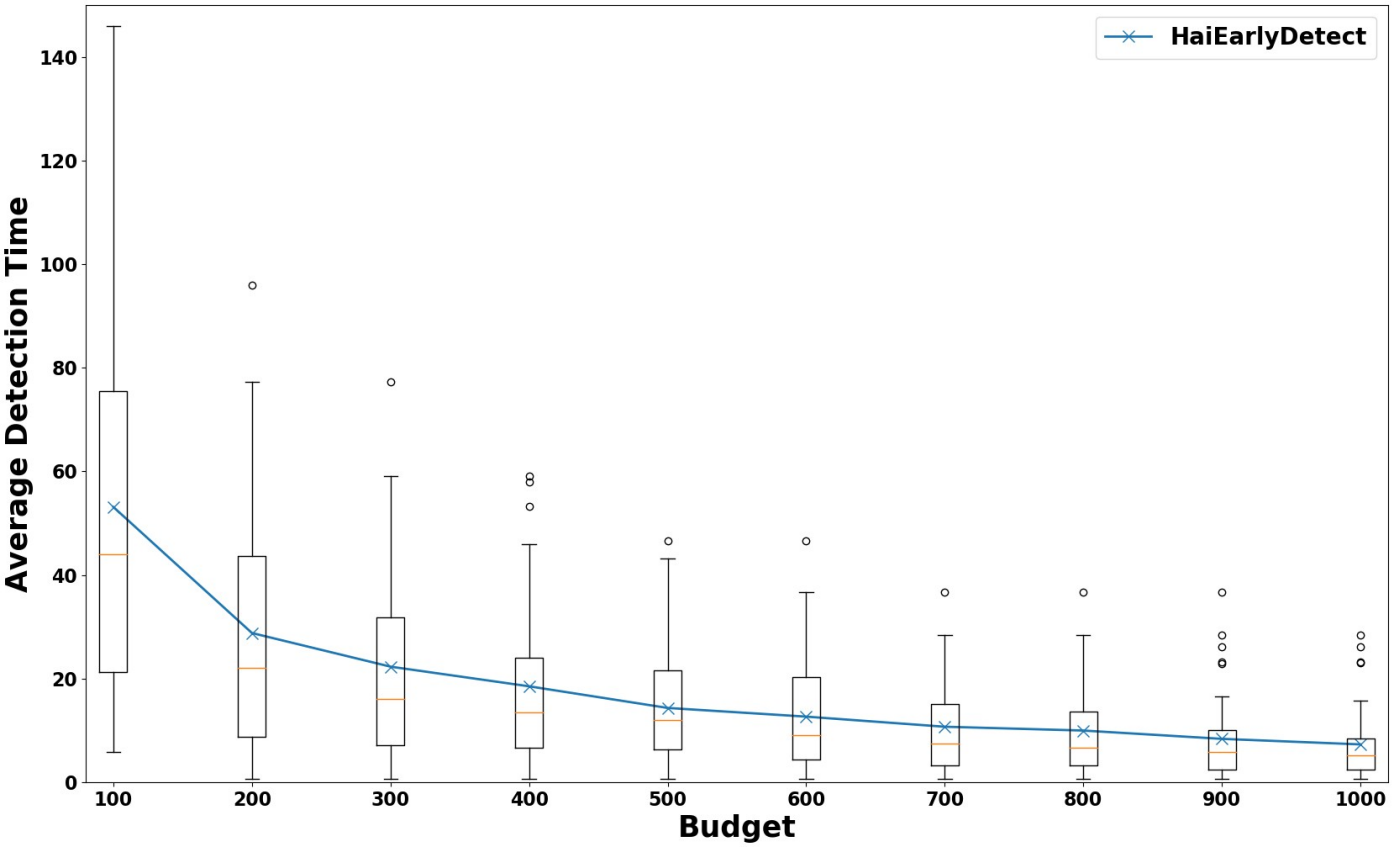
Variation in detection time (in days) for various budgets. As budget increases, the variation decreases.

A desirable property of sensors is that they aid in early detection of outbreaks. Here we study the average detection time of future outbreaks using the sensors and rates selected by HaiEarlyDetect. In this experiment, we first divided our simulations into equally sized training and testing sets, each having 100 simulations. We ran HaiEarlyDetect on the training set to detect sensors and rates at which to monitor them. Then, we monitored the selected sensors at the inferred rates and measured the detection time for each simulation in the testing set. We repeated the entire process for various budgets. The detection time averaged over 100 simulated outbreaks in the testing set is summarized in Fig 9 and the variance in the detection time is shown in Fig 10.

As shown in the figure, as the budget increases the average detection time decreases. According to our results, the average time to detect an outbreak in the testing set while monitoring sensors selected for budget of 1000 is roughly six days. This is impressive considering the fact that monitoring all agents results in detection time of 4 days monitoring all of more than 1200 nurses results in detection time of 8 days. Hence, monitoring these sensors detect the outbreak earlier with fewer budget. Another advantage of our sensors is that they are diverse. Significant proportion of the selected sensors include patients and fomites, which are easier to monitor than the nurses. Hence, monitoring the sensors selected by HaiEarlyDetect also has an economic advantage.

An interesting observation seen in Fig 10 is that the variablity in average detection time decreases with the increase in budget. Hence, we expect the performance of our sensors to be fairly consistent in detecting future outbreaks for larger budgets. Moreover, the median time to detect an outbreak (as shown by the box plots) is always less than the average. Hence, we expect that performance of HaiEarlyDetect to be generally better than that suggested by the average detection time. For budget of 1000, the median detection time is just 5 days. Note that monitoring all agents results in detection time of 4 days. This implies that in practice our approach requires only 1000 swabs per day to detect an outbreak within a single day of the first infection.

### Number of cases prevented

An interesting question is how many potential cases can be prevented by monitoring the sensors selected by HaiEarlyDetect. Here we study how many nodes get infected before an outbreak is detected and how many potential infections can be prevented by monitoring our sensors for various budgets. As in the previous experiment, for a given budget, we leverage 100 simulations to select sensors and their monitoring rates. Once the sensors are selected, we count the number of infections that occur in a test simulation before a sensor is infected and how many further infections occur following the infections of sensors. We then average these numbers over 100 test simulations. The results are summarized in Table 2.

**Table 2:** Average number of infections (± standard deviation) before an outbreak that is detected by monitoring sensors selected by HaiEarlyDetect and potential number of infections prevented by detection the outbreak. For a budget of 1000 roughly 77% of potential cases are prevented.

Budget	# infections when detected	# potential infections prevented
10	17.1 ± 5.8	4.3 ± 4.6
50	13.9 ± 5.9	6.5 ± 4.2
100	11.3 ± 4.4	8.2 ± 3.9
200	6.5 ± 0.5	15.0 ± 3.3
500	6.0 ± 0.2	16.2 ± 3.3
1000	5.4 ± 0.0	17.0 ± 3.2

As shown in Table 2, for the budget of 10 samples/swabs, 4.31 potential future infections could prevented. Note that there are only 23 infections on average per simulation. For the budget of only 200, 15.02 infections could be prevented, which is about 66% of potential infections. The number goes up to 17, or 74% for the budget of 1000. The result shows that even for a low budget (less than 200 swabs per day), our approach could help prevent a significant number of future infections.

### Qualitative distribution of sensors

Next we study the types of agents that are selected by HaiDetect as sensors. For this experiment, we use 100 randomly selected simulations to detect sensors for a wide range of budgets. After the sensors are selected, we sum up the rates of each category of agents like nurses, doctors, patients, and so on.

**Fig 11:**
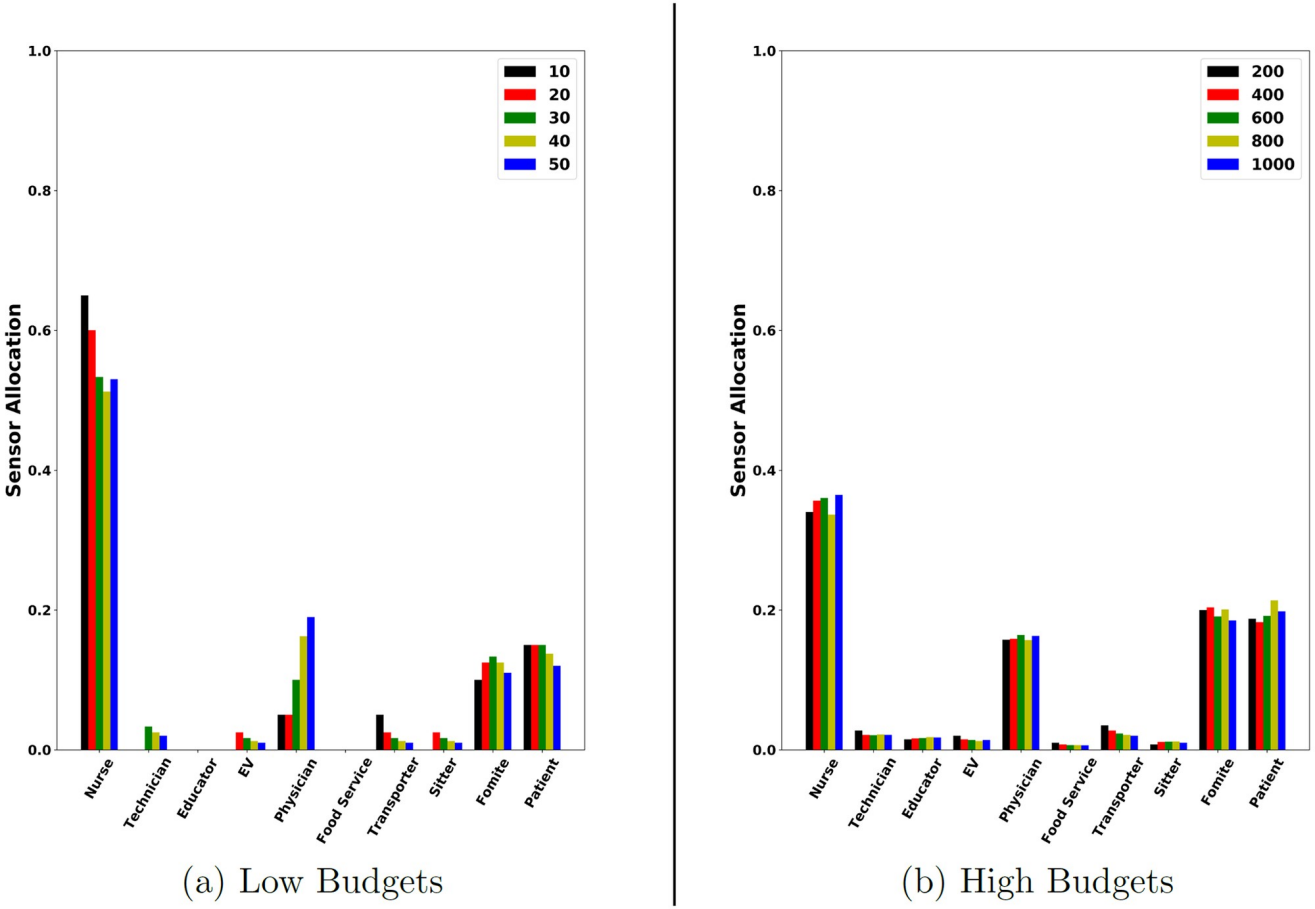
Variation of sensor set distribution for HaiDetect with budget. HaiDetect selects intuitively meaningful sensors even for lower budgets.

Fig 11(a) shows the distribution of sensor allocation for each category of agents at low budgets. We observe that for a budget of 10, nearly 60% of the total budget is spent on selecting nurses. Since nurses are the most mobile agents, the result highlights the fact that HaiDetect selects the most important agents as sensors early on. Similarly, Fig 11 (b) shows the distribution of sensors for higher budgets. Here we observe that nearly 35% of the budget is allocated for nurses. Fomites and patients have roughly equal allocations of about 20%. 17% of the budget is allocated to doctors. The rest of the categories have minimal allocation. The distribution shows that HaiDetect selects heterogeneous sensors including both people and objects/locations as intended.

**Fig 12:**
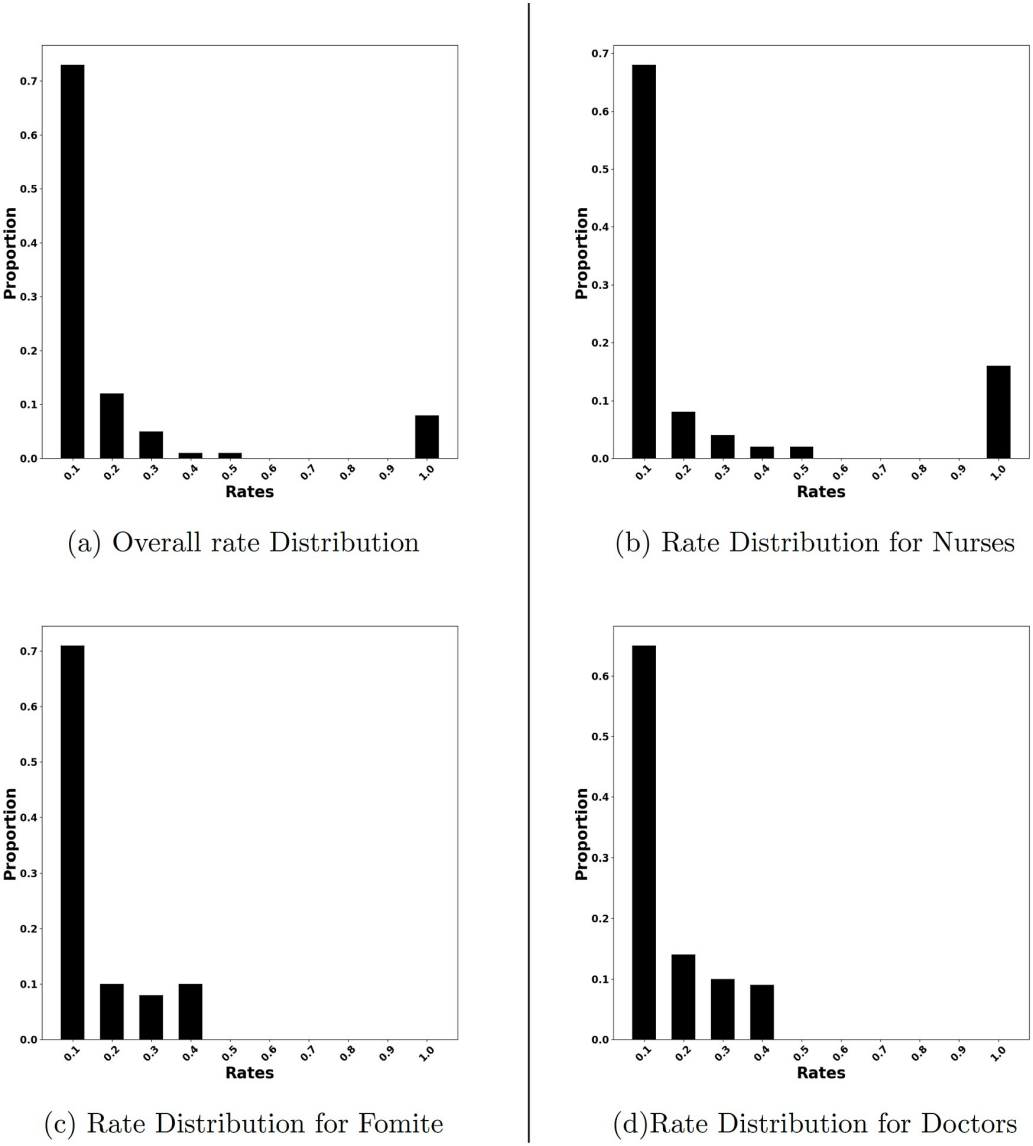
Distribution of allocation for different rates. Since most of the sensors selected by HaiDetect have low rates, they have to be monitored only sporadically.

Finally, we are also interested on the scheduling implications of the sensors selected by HaiDetect. To this end, we measure the aggregated proportion of budget assigned to each rate for the sensors we select. The results are summarized in Fig 12. As shown in Fig 12(a), most of the sensors have rate of 0.1. Very few sensors have rate from 0.2 to 0.5. Finally, there is a sudden spike at rate = 1.0. When we look at rate distribution for each category separately, interestingly we observe that only nurses have rates of 1.0. This implies that certain nurses have to be monitored each day to detect HAI outbreak. The reason behind this unexpected behaviour can be attributed to the fact that the hospital from where the mobility log was collected, required all the nurses to attend a daily meeting. Hence, all the nurses were in contact with each other every day and it is likely that nurses infect each other in case of an outbreak. Hence, there is an advantage in monitoring some of the nurses everyday to quickly detect HAI outbreak.

## Discussion

Our results show that our sensor sets have good performance with respect to both the detection probability and detection time objectives, compared to the Celf baseline, as well as other natural heuristics motivated by standard practices in a hospital. A relatively low budget ensures a high detection probability, with low variance. Further, we find that training with a small set of simulation cascades (e.g., 20) is able to ensure reasonably high detection probability (about 0.8). This suggests the strategy is quite practical, since, in general, detailed knowledge of how HAIs cascade through a hospital system are difficult to obtain, thus motivating this agent-based modeling approach.

It is important to note that for simplicity of interpretation, we assume perfect detection at each sensor, thus these results to provide an estimate of best case performance in the real world. The sensitivity and specificity of detecting *C. difficile* on different fomites varies widely and the stochastic nature of this process would require extensive sensitivity analyses, which is beyond the scope of this particular study. Additionally, we did not simulate the course of care for patients in this simulation, which is also highly stochastic, thus we did not include detection outside of the designed sensor scheme. This assumption removes clinically appropriate detections, which would further improve the probability of detections reduce the time to detection.

An area for improvement of the algorithm is the lengthy time of detection anticipated for limited budgets under 100 sensors. A much larger budget is needed for quick detection times, which is a harder problem for myriad reasons. In particular, a budget of about 1000 is needed to get the detection time under a week. In contrast, monitoring strategies which monitor all agents of a specific type (e.g., all patients, all technicians, all nurses) lead to much lower detection time bounds. However, the number of nurses is almost 20% larger than this budget; further, monitoring all nurses is not a very practical strategy. Our sensor sets are much more practical in the sense that they include different types of agents (including fomites). Only when all nurses are monitored, is the detection time is close to a week. It is important to note that this time to detection is being measured from the very beginning of infection from the first case in the cascade. Many HAI infection cascades can remain undetected for many generations of transmission, which is impossible to measure in the real-world, and indeed may persist in a hospital for years. Thus a monitoring system that increases the probability of eventually detecting a cascade and provides expected detection times in the order of several months can represent a significant improvement.

Our approach requires mobility and activity data in a hospital, along with data on disease incidence. This type of data are difficult to obtain even well resourced field trials, especially in the quantity needed for the robust sensitivity analyses presented here. While these limitations were the primary motivation for the use of agent-based simulations, this simulated data remains a major limitation. While a field trial would provide more convincing evidence of the actual real-world performance of HaiDetect, the purpose here was to evaluate its performance and motivate its potential use in a resource intensive field trial. We also note that no prior work on HAI modeling and control studies consider problems at the level of detail we consider here.

Our approach shows how HAI outbreaks can be detected in a resource intensive case and provides a way to prioritize detection efforts which are useful from practitioner’s viewpoint. Based on our empirical results, we recommend monitoring nurses, physicians, objects/locations and patients. These results are generalizable to settings like most ICU units in the United States that follow similar divisions of labor and intensity of care. To customize the approach to other venues, we recommend practitioners to follow our calibration and simulation approach to produce simulated outbreaks using similar data, which would provide better estimates on the exact reductions one could expect and which exact individuals and locations to monitor. Similarly, detecting HAI as early as possible is important and quite costly when not detected. Our results show that using 1000 swabs a day, the infection could be detected with a delay of a single day. With a budget for 200 swabs a day we could detect the outbreak within 16 days on average. Practitioners can use our results or methodology, to weigh the costs and benefits appropriate to their location based on their resources and desired need for timeliness and cases prevented.

Our results on sensor rate distribution and comparison against the CELF show the benefits of monitoring with lower rates on a larger set rather than higher rates on a smaller set. This can be more robust in practice as compliance tends to fade when focused with high intensity on a smaller set of individuals. Similarly, with a budget of 50 swabs a day, we can detect future outbreaks with a success rate close to of 1. And with a budget of 200 swabs a day, we can detect the outbreak within 16 days on average. This is quite surprising and useful for practitioners. And finally, with a budget of around 200 swabs a day, the outbreak can be detected before 70% of the infections occur. Hence, this approach can also aid in control of HAI outbreak. From a practitioner’s perspective prevention of this level of potential future cases is a great benefit.

### Conclusion

Effective and early detection of HAI outbreaks are important problems in hospital infection control, and have not been studied systematically so far. While these are challenging problems, understanding their structure can help in designing effective algorithms and optimizing resources. Current practices in hospitals are fairly simple, and do not attempt to optimize resources. Our algorithms perform better than many natural heuristics, and our results show that a combination of data and model driven approach is effective in detecting HAIs. Since there is limited data on disease incidence, good models and simulations play an important role in designing algorithms and evaluating them.

## Supporting information

S1 Text Appendix: Proofs for submodularity, NP-hardness, and online bound. (PDF)

S1 Fig Variation of sensor set distribution for Celf. Note that Celf picks more patients and less nurses than HaiDetect for lower budgets, which explains its poor performance in the test set (Fig 6).
(TIF)

S2 Fig Average detection time on training data for HaiEarlyDetect. As expected, HaiEarlyDetect has even better detection time on training data.
(TIF)

S3 Fig Variation in average detection time on training data for HaiEarlyDetect. (TIF)

S4 Fig Overlap between sensors selected by HaiDetect and Celf various budgets. There is only roughly 10% overlap between Celf and HaiDetect, highlighting that HaiDetect selects different nodes and rates than Celf.
(TIF)

S1 Data Mobility logs for days 1 to 100. (ZIP)

S2 Data Mobility logs for days 101 to 200. (ZIP)

S3 Data Calibrated simulations of *C. difficile* outbreak. (ZIP)

S4 Data Social network resulted by the interactions in the mobility log. (ZIP)

S5 Data A documentation of the data. Describes the format of each file in detail.
(MD) Click here for additional data file.

## References

[1] Stone P. W., “Economic burden of healthcare-associated infections: an american perspective,” *Expert review of pharmacoeconomics & outcomes research*, vol. 9, no. 5, pp. 417–422, 2009 10.1586/erp.09.53 19817525 PMC2827870

[2] Cassini A., Plachouras D., Eckmanns T., Sin M. A., Blank H.-P., Ducomble T., Haller S., Harder T., Klingeberg A., Sixtensson M., et al, “Burden of six healthcare-associated infections on european population health: estimating incidence-based disability-adjusted life years through a population prevalence-based modelling study,” *PLoS medicine*, vol. 13, no. 10, p. e1002150, 2016 10.1371/journal.pmed.1002150 27755545 PMC5068791

[3] Leclère B., Buckeridge D. L., Boëlle P.-Y., Astagneau P., and Lepelletier D., “Automated detection of hospital outbreaks: A systematic review of methods,” *PloS one*, vol. 12, p. e0176438, 4 2017 10.1371/journal.pone.0176438 28441422 PMC5404859

[4] Lewis B., Eubank S., Abrams A. M., and Kleinman K., “In silico surveillance: evaluating outbreak detection with simulation models.,” *BMC medical informatics and decision making*, vol. 13, p. 12, 1 2013 10.1186/1472-6947-13-12 23343523 PMC3691709

[5] Lofgren E. T., Moehring R. W., Anderson D. J., Weber D. J., and Fefferman N. H., “A mathematical model to evaluate the routine use of fecal microbiota transplantation to prevent incident and recurrent clostridium difficile infection,” *Infection Control and Hospital Epidemiology*, 2013. 10.1086/674394 PMC3977703 24334794

[6] Lofgren E., Cole S., Weber D., Anderson D., and Moehring R., “Hospital-acquired clostridium difficile infections: Estimating all-cause mortality and length of stay,” *Epidemiology*, pp. 570–75, 2014 10.1097/EDE.0000000000000119 24815305 PMC4224274

[7] Cosgrove S.E., Qi Y., Kaye K., Harbarth S., Karchmer A., and Carmeli Y., “The impact of methicillin resistance in staphylococcus aureus bacteremia on patient outcomes: Mortality, length of stay and hospital charges,” *Infection Control and Hospital Epidemiology*, pp. 166–174, 2005. 15756888 10.1086/502522

[8] Klevens R. M., Morrison M., Nadle J., Gershman K., Ray S., Harrison L., Lynfield R., Dumyati G., Townes J., Craig A., Zell E., Fosheim G., McDougal L., Carey R., and Fridkin S., “Invasive methicillin-resistance staphylococcus aureus infections in the united states,” *JAMA*, pp. 1763–71, 2007 10.1001/jama.298.15.1763 17940231

[9] van Kleef E., Robotham J. V., Jit M., Deeny S. R., and Edmunds W. J., “Modelling the transmission of healthcare associated infections: a systematic review,” *BMC infectious diseases*, vol. 13, p. e1001172, 6 2013 10.1186/1471-2334-13-294 PMC3701468 23809195

[10] J. Leskovec, A. Krause, C. Guestrin, C. Faloutsos, J. VanBriesen, and N. Glance, “Cost-effective outbreak detection in networks,” in *Proceedings of the 13th ACM SIGKDD international conference on Knowledge discovery and data mining*, pp. 420–429, ACM, 2007.

[11] Christakis N. A. and Fowler J. H., “Social network sensors for early detection of contagious outbreaks,” *PloS one*, vol. 5, no. 9, p. e12948, 2010 10.1371/journal.pone.0012948 20856792 PMC2939797

[12] H. Shao, K. Hossain, H. Wu, M. Khan, A. Vullikanti, B. A. Prakash, M. Marathe, and N. Ramakrishnan, “Forecasting the flu: designing social network sensors for epidemics,” *SIGKDD epiDAMIK Workshop*, 2018.

[13] Reis B. Y., Kohane I. S., and Mandl K. D., “An epidemiological network model for disease outbreak detection,” *PLoS medicine*, vol. 4, no. 6, p. e210, 2007 10.1371/journal.pmed.0040210 17593895 PMC1896205

[14] Y. Zhang and B. A. Prakash, “Dava: Distributing vaccines over networks under prior information,” in *Proceedings of the 2014 SIAM International Conference on Data Mining*, pp. 46–54, SIAM, 2014.

[15] L. Briesemeister, P. Lincoln, and P. Porras, “Epidemic profiles and defense of scale-free networks,” in *Proceedings of the 2003 ACM workshop on Rapid malcode*, pp. 67–75, ACM, 2003.

[16] Zhang Y., Adiga A., Saha S., Vullikanti A., and Prakash B. A., “Near-optimal algorithms for controlling propagation at group scale on networks,” *IEEE Transactions on Knowledge and Data Engineering*, vol. 28, no. 12, pp. 3339–3352, 2016 10.1109/TKDE.2016.2605088

[17] P. Rozenshtein, A. Gionis, B. A. Prakash, and J. Vreeken, “Reconstructing an epidemic over time,” in *Proceedings of the 22nd ACM SIGKDD International Conference on Knowledge Discovery and Data Mining*, pp. 1835–1844, ACM, 2016.

[18] J. M. Jimenez, B. L. Lewis, and S. Eubank, “The application of macroergonomics and simulation to improve control of healthcare acquired infections,” in *Proceedings of the 2013 Winter Simulation Conference: Simulation: Making Decisions in a Complex World*, pp. 3938–3939, IEEE Press, 2013.

[19] J. M. Jiménez, B. Lewis, and S. Eubank, “Hospitals as complex social systems: Agent-based simulations of hospital-acquired infections,” in *International Conference on Complex Sciences*, pp. 165–178, Springer, 2012.

[20] J. M. Jiménez, *The utilization of macroergonomics and highly-detailed simulation to reduce healthcare-acquired infections*. PhD thesis, Virginia Tech, 2014.

[21] Rubin M. A., Jones M., Leecaster M., Khader K., Ray W., Huttner A., Huttner B., Toth D., Sablay T., Borotkanics R. J., et al, “A simulation-based assessment of strategies to control clostridium difficile transmission and infection,” *PloS one*, vol. 8, no. 11, p. e80671, 2013 10.1371/journal.pone.0080671 24278304 PMC3836736

[22] Nelson R. E., Jones M., Leecaster M., Samore M. H., Ray W., Huttner A., Huttner B., Khader K., Stevens V. W., Gerding D., et al, “An economic analysis of strategies to control clostridium difficile transmission and infection using an agent-based simulation model,” *PloS one*, vol. 11, no. 3, p. e0152248, 2016 10.1371/journal.pone.0152248 27031464 PMC4816545

[23] R. Carrico, K. Bryant, F. Lessa, B. Limbago, L. Fauerbach, J. Marx, F. Sands, D. Stephens, K. Westhusing, and T. Wiemken, “Guide to preventing clostridium difficile infections,” *APIC*, vol. 16, 2013.

[24] Cohen S. H., Gerding D. N., Johnson S., Kelly C. P., Loo V. G., McDonald L. C., Pepin J., and Wilcox M. H., “Clinical practice guidelines for clostridium difficile infection in adults: 2010 update by the society for healthcare epidemiology of america (shea) and the infectious diseases society of america (idsa),” *Infection Control & Hospital Epidemiology*, vol. 31, no. 5, pp. 431–455, 2010 10.1086/651706 20307191

[25] Karadsheh Z. and Sule S., “Fecal transplantation for the treatment of recurrent clostridium difficile infection,” *North American journal of medical sciences*, vol. 5, no. 6, p. 339, 2013 10.4103/1947-2714.114163 23923106 PMC3731863

[26] Sunenshine R. H., McDonald L. C., et al, “Clostridium difficile-associated disease: new challenges from an established pathogen,” *Cleveland Clinic journal of medicine*, vol. 73, no. 2, p. 187, 2006. 16478043 10.3949/ccjm.73.2.187

[27] Yoo J. and Lightner A. L., “Clostridium difficile infections: what every clinician should know,” *The Permanente Journal*, vol. 14, no. 2, p. 35, 2010 10.7812/TPP/10-001 20740115 PMC2912081

[28] C. L. Barrett, K. R. Bisset, S. G. Eubank, X. Feng, and M. V. Marathe, “Episimdemics: an efficient algorithm for simulating the spread of infectious disease over large realistic social networks,” in *High Performance Computing, Networking, Storage and Analysis, 2008. SC 2008. International Conference for*, pp. 1–12, IEEE, 2008.

[29] Halloran M. E., Ferguson N. M., Eubank S., Longini I. M., Cummings D. A., Lewis B., Xu S., Fraser C., Vullikanti A., Germann T. C., et al, “Modeling targeted layered containment of an influenza pandemic in the united states,” *Proceedings of the National Academy of Sciences*, vol. 105, no. 12, pp. 4639–4644, 2008 10.1073/pnas.0706849105 PMC2290797 18332436

[30] Wendelsdorf K. V., Alam M., Bassaganya-Riera J., Bisset K., Eubank S., Hontecillas R., Hoops S., and Marathe M., “Enteric immunity simulator: a tool for in silico study of gastroenteric infections,” *IEEE transactions on nanobioscience*, vol. 11, no. 3, pp. 273–288, 2012 10.1109/TNB.2012.2211891 22987134 PMC3715318

[31] J.-S. Yeom, A. Bhatele, K. Bisset, E. Bohm, A. Gupta, L. V. Kale, M. Marathe, D. S. Nikolopoulos, M. Schulz, and L. Wesolowski, “Overcoming the scalability challenges of epidemic simulations on blue waters,” in *2014 IEEE 28th International Parallel and Distributed Processing Symposium*, pp. 755–764, IEEE, 2014.

[32] T. Soma and Y. Yoshida, “Maximizing monotone submodular functions over the integer lattice,” in *International Conference on Integer Programming and Combinatorial Optimization*, pp. 325–336, Springer, 2016.

