# Supplementary material for: Fast and near-optimal monitoring for healthcare acquired infection outbreaks
Source: PLoS Comput Biol. 2019 Sep 16;15(9):e1007284. doi: 10.1371/journal.pcbi.1007284 (PMC6762212; doi:10.1371/journal.pcbi.1007284)
Supplement: S1 Text — (PDF) [file pcbi.1007284.s001.pdf]

Appendix: Fast and Near-Optimal Monitoring for Healthcare Acquired Infection Outbreaks

Bijaya Adhikari[1*], Bryan Lewis[2†], Anil Vullikanti[2,3†], José Mauricio Jiménez[4§] and B. Aditya Prakash[1*]

1 Department of Computer Science, Virginia Tech, Blacksburg, VA, USA
2 Biocomplexity Institute & Initiative, University of Virginia, Charlottesville, VA, USA
3 Department of Computer Science, University of Virginia, Charlottesville, VA, USA
4 Department of Systems Engineering, United States Military Academy, West Point, NY, USA

∗ {bijaya, badityap}@cs.vt.edu

NP-hardness Proofs

Here we show that both of our problems are NP-hard. Recall that the first problem we solve is as follows:

Problem 1. *Find the vector* $\mathbf{r}^*$*, such that*

$$\mathbf{r}^* = \arg\max_{\mathbf{r}} \sum_{i \in \mathcal{I}} P(i|\mathbf{r}) \tag{1}$$

and $\sum_v \mathbf{r}[v] \cdot \mathbf{c}[v] \leq B$.

Now, we prove the following lemma.

Lemma 1. *Problem 3 is NP-hard.*

Proof. Here we show that NP-Complete SetCover problem reduces to Problem 3 in polynomial time. We do this by showing that the SetCover problem can be seen as special case of Problem 3, where entries in the vector $\mathbf{r}$ can only take 0 or 1 values.

Consider an arbitrary instance of the NP-complete SetCover problem (decision version) with universal set $U = \{u_1, u_2, \ldots, u_n\}$, subsets $S_1, S_2, \ldots, S_m$ where the goal is to determine whether there exists at most k subsets whose union equals the universal set U.

Given an arbitrary instance of the SetCover problem, we construct Problem 3 instance as follows:

- For each element $u_i \in U$, construct a simulation instance $C_i \in I$.
- To each $C_i \in I$ corresponding to $u_i \in U$, add all subset S_j such that $u_i \in S_j$.
- Set budget $B = k$.
- Set cost of each S_k to be 1.

Now, deciding whether there exists at most k subsets whose union equals U is equivalent to deciding whether there exists $\mathbf{r}^*$ such that $\sum_{i \in \mathcal{I}} P(i|\mathbf{r}^*) = |U|$. First we show that, if there are k subsets whose union equals U, then there is $\mathbf{r}^*$, such that $\sum_{i \in \mathcal{I}} P(i|\mathbf{r}^*) = |U|$.

Let X be the set of k subsets whose union equals U. Note that by construction each simulation instance $C_i \in I$ corresponding to $u_i \in U$, consists of all subset S_j such that $u_i \in S_j$. Now since, union of subsets in X equals U, there must be atleast one $S_x \in X$ in each of C_i (Otherwise, the union of subsets in X will not be equal to U). Now, one can assign $\mathbf{r}[S_x] = 1$ for each of these k subsets in X. Note that since there are k subsets, only k entries in $\mathbf{r}[S_x]$ are set to 1 and rest are set to 0. Hence, $\sum_{S_x \in \mathbf{r}} C(S_x)\mathbf{r}[S_x] = B$. Since, at least one S_x appears in each C_i, $P(C_i|\mathbf{r}) = 1$ for all the C_is. Since there are $|U|$ C_i, $\sum_{i \in \mathcal{I}} P(i|\mathbf{r}^*) = |U|$.

Now, we show that if there exists $\sum_{i \in \mathcal{I}} P(i|\mathbf{r}^*) = |U|$, then there must be at least k subsets whose union equals U. Since, $\sum_{i \in \mathcal{I}} P(i|\mathbf{r}^*) = |U|$, $P(C_i|\mathbf{r}) = 1$ for each C_i (Otherwise, sum would be less than $|U|$). Since, the budget $B = k$, there must be at most k S_x, such that $\mathbf{r} * [S_x] = 1$, let the set of such S_x be X. By construction, S_x is included in C_i if and only if $u_i \in S_x$. Now, since $P(C_i|\mathbf{r}) = 1$ for each C_i, each C_i has atleast one $S_x \in X$, which implies, union of all the $S_x \in X = U$.

Hence, Problem 3 is NP-hard. □

The second problem we solve is as follows:

Problem 2. *Find the vector* $\mathbf{r}^*$*, such that*

$$\mathbf{r}^* = \arg\max_{\mathbf{r}} \sum_{i \in \mathcal{I}} [T_{max} - D(i|\mathbf{r})] \tag{2}$$

and $\sum_v \mathbf{r}[v] \cdot \mathbf{c}[v] \leq B$*.*

Now we prove the following lemma.

Lemma 2. *Problem 2 is NP-hard.*

Proof. We use similar strategy as in the Proof of Lemma 1 to show that Problem 2 is NP-hard. We still consider the special case of Problem 2, where the where entries in the vector $\mathbf{r}$ can only take 0 or 1 values. Consider the same instance of SetCover problem as above.

Given an arbitrary instance of the SetCover problem, we construct Problem 3 instance as follows:

- For each element $u_i \in U$, construct a simulation instance $C_i \in I$.
- To each $C_i \in I$ corresponding to $u_i \in U$, add all subset S_j such that $u_i \in S_j$, set the earliest time of infection for all S_j to be zero, i.e., $0 \in \gamma(S_j, C_i)$ for all $(S_j, C)i)$ pairs.
- Set budget $B = k$.
- Set cost of each S_k to be 1.

Following similar argument as above, we can show that deciding whether there exists at most k subsets whose union equals U is equivalent to deciding whether there exists $\mathbf{r}^*$ such that $\sum_{i \in \mathcal{I}} P(i|\mathbf{r}^*) = |U| \times T_{max}$ □

Submodularity Proof

The first problem we solve asks to optimize the probability of detection. Problem 1 is an optimization problem defined over a continuous space of rate vectors $X \in [0, 1]^n$, and is NP-hard to solve optimally. We show here that it has some useful structural properties, which make it amenable to good approximations. We start with some definitions.

Definition 1. *(Vector inequality) For* $\mathbf{a}, \mathbf{b} \in X$*,* $\mathbf{a} \leq \mathbf{b}$ *implies that* $\forall_i,\ \mathbf{a}[i] \leq \mathbf{b}[i]$.

Definition 2. *(Submodularity) A function* $f : X \to R^{\geq 0}$ *is submodular iff for all* $\mathbf{a}, \mathbf{b} \in X$*,* $f(\mathbf{a}) + f(\mathbf{b}) \geq f(\mathbf{a} \vee \mathbf{b}) + f(\mathbf{a} \wedge \mathbf{b})$*, where* $\mathbf{a} \vee \mathbf{b}$ *and* $\mathbf{a} \wedge \mathbf{b}$ *are element-wise maxima and minima of* $\mathbf{a}$ *and* $\mathbf{b}$.

This is an extension of the more commonly used notion of submodularity of functions defined on sets. Such functions can be maximized using a greedy algorithm.

Submodularity

Our goal here is to prove that the objective in Problem 1 is both monotone and submodular. Let $f(\mathbf{r}) = \sum_{i \in \mathcal{I}} f_i(\mathbf{r})$, where $f_i(\mathbf{r}) = P_d(i|\mathbf{r})$. It is obvious that $f_i(\mathbf{r})$ is monotone, which implies f is monotone. We next consider its submodularity.

Lemma 3. *f_i is submodular.*

Proof. Recall that $P_d(i|\mathbf{r}) = 1 - \prod_{v \in P \cup L} (1 - \mathbf{r}[v])^{\tau(v,i)}$, where $\tau(v, i)$ is the number of days in which node v is in infected state in simulation instance i. Let $g_i = \prod_{v \in P \cup L} \prod_{k=0}^{\tau(v,i)} (1 - \mathbf{r}[v])$. We will show that g_i is supermodular, which will imply that $f_i = 1 - g_i$ is submodular.

Note that $\tau(v, i)$ is constant for each simulation i. Hence, $g_i(\mathbf{r})$ can be rewritten as $\prod_{v \in P \cup L} \phi(\mathbf{r}[v])$.

We consider a fixed i. Let $\alpha = g_i(\mathbf{x} \wedge \mathbf{y})$, $\beta = \frac{g_i(\mathbf{x})}{\alpha}$, and $\gamma = \frac{g_i(\mathbf{y})}{\alpha}$

$$
\begin{aligned}
g_i(\mathbf{x} \vee \mathbf{y}) &= \prod_{v \in P \cup L} \phi(\max\{\mathbf{x}[v], \mathbf{y}[v]\}) \\
&= \frac{\prod_{v \in P \cup L} \phi(\mathbf{x}[v]) \prod_{v \in P \cup L} \phi(\mathbf{y}[v])}{\prod_{v \in P \cup L} \phi(\min\{\mathbf{x}[v], \mathbf{y}[v]\})} \\
&= \frac{g_i(\mathbf{x}) g_i(\mathbf{y})}{g_i(\mathbf{x} \wedge \mathbf{y})} \\
&= \alpha\beta\gamma \qquad (3)
\end{aligned}
$$

Now,

$$
\begin{aligned}
& g_i(\mathbf{x} \vee \mathbf{y}) + g_i(\mathbf{x} \wedge \mathbf{y}) - (g_i(\mathbf{x}) + g_i(\mathbf{y})) \\
&= \alpha\beta\gamma + \alpha - (\alpha\beta + \alpha\gamma) \\
&= \alpha(\beta\gamma + 1 - \beta - \gamma) \\
&= \alpha(1 - \beta)(1 - \gamma) \\
&\geq 0 \qquad (4)
\end{aligned}
$$

Hence, $g_i(\mathbf{x} \vee \mathbf{y}) + g_i(\mathbf{x} \wedge \mathbf{y}) \geq g_i(\mathbf{x}) + g_i(\mathbf{y})$, which implies g_i is supermodular and f_i is submodular. Finally, any linear combination of submodular function is also submodular, which implies $f()$ is submodular. □

Online Bound

Here we discuss the online bound for our problem on maximizing detection likelihood (Problem 1). Let bold small letters indicate vectors. Similarly, for any given vector $\mathbf{a}$, let $\mathbf{a}_v$ denote the vth entry of $\mathbf{a}$.

Note that our problem asks to find a rate vector r indicating monitoring rate for each human agents and location (static agents) in the given mobility log $G(P, L, E, T)$.

As in the paper, $c(v)$ represents the cost of monitoring the node v. Recall that the problem is formally defined as follows:

Problem 3. *Find the vector* $\mathbf{r}^*$*, such that*

$$\mathbf{r}^* = \arg\max_{\mathbf{r}} \sum_{i \in \mathcal{I}} P(i|\mathbf{r}) \tag{5}$$

and $\sum_v \mathbf{r}[v] \cdot \mathbf{c}[v] \leq B$.

The objective in Equation 5 for Problem 3 is a monotone sub-modular function (See Appendix 1). Here we give a bound on how far our solution is from the optimal for any given budget B. Now, let us start with some notations and definitions:

Definition 3. *(Vector inequality) For* $\mathbf{a}, \mathbf{b} \in X$*,* $\mathbf{a} \leq \mathbf{b}$ *implies that* $\forall_i,\ \mathbf{a}[i] \leq \mathbf{b}[i]$.

Definition 4. *(Element-wise Maxima and Minima)* $\mathbf{a} \vee \mathbf{b}$ *and* $\mathbf{a} \wedge \mathbf{b}$ *are element-wise maxima and minima of* $\mathbf{a}$ *and* $\mathbf{b}$.

Definition 5. *(Characteristic Vector) Characteristic vector* χ^S *of a set* S *is a vector such that* $\chi^S_v = 1$ *if* $v \in S$*,* 0 *otherwise.*

Definition 6. *(Positive Support) For a given rate vector* $\mathbf{a}$*, its positive support* $supp^+(\mathbf{a})$ *is a set of elements* v *such that* $\mathbf{a}_v > 0$.

Let the current rate vector given by our approach for Problem 1 for a budget B be $\hat{\mathbf{r}}$. Similarly, let the optimal rate vector for the same budget be $\mathbf{r}^*$.

For all nodes v and for $a \in [0, 1]$, let us define Δ_v as follows:

$$\Delta_v = \max_a \Big[R(\hat{\mathbf{r}} \vee a \cdot \chi^{\{v\}}) - R(\hat{\mathbf{r}})\Big] \tag{6}$$

Similarly let us define σ_v as the argument which maximizes Δ_v

$$\sigma_v = \arg\max_a \Big[R(\hat{\mathbf{r}} \vee a \cdot \chi^{\{v\}}) - R(\hat{\mathbf{r}})\Big] \tag{7}$$

Note that for a node $v \in supp^+(\hat{\mathbf{r}})$, σ_v can only be greater than $\hat{\mathbf{r}}_v$.

Now, let $\delta_v = \frac{\Delta_v}{c(v) \cdot \sigma_v}$. Note that for each node v, there is a single δ.

Let the sequence of nodes $s_1, s_2, \ldots, s_n$ be ordered in decreasing order of δ_v.

Now let K be the index such that $\theta = \sum_{i=1}^{K-1} c(s_i)\sigma_{s_i} \leq B$ and $\sum_{i=1}^{K} c(s_i)\sigma_{s_i} > B$. Now the online bound for the current rate $\hat{\mathbf{r}}$ assigned by our algorithm is as follows:

Lemma 4. *Online Bound.*

$$R(\mathbf{r}^*) \leq R(\hat{\mathbf{r}}) + \sum_{i=1}^{K-1} \Delta s_i + \frac{B - \theta}{c(s_K)\sigma_{s_K}} \Delta_{s_K}$$

Proof. First of all we have,

$$R(\mathbf{r}^*) \leq R(\hat{\mathbf{r}} \vee \mathbf{r}^*) \tag{8}$$

This is due to the monotonocity of R. Now, since R is a submodular lattice function, following holds,

$$R(\hat{\mathbf{r}} \vee \mathbf{r}^*) \leq R(\hat{\mathbf{r}}) + \sum_{v \in supp^+(\mathbf{r}^* - \hat{\mathbf{r}})} \Big[R(\hat{\mathbf{r}} \vee \mathbf{r}^*_v \cdot \chi^{\{v\}}) - R(\hat{\mathbf{r}})\Big] \tag{9}$$

Let $\mathbf{r}'$ be the vector such that $\mathbf{r}'_v = \max(\mathbf{r}^*_v - \hat{\mathbf{r}}_v, 0)$. Basically, $\mathbf{r}'$ is the vector representation of $supp^+(\mathbf{r}^* - \hat{\mathbf{r}})$. Note that $\hat{\mathbf{r}}$ and $\mathbf{r}^*$ both satisfy the budget constraints

as they are feasible solutions to Problem 1. Hence, $\mathbf{r}'$ also satisfies the budget constraint (as $\mathbf{r}'_v$ cannot be greater than $\mathbf{r}^*_v$ for any v). Hence, we have the following:

$$\sum_{v \in supp^+(\mathbf{r}')} \left[R(\hat{\mathbf{r}} \vee \mathbf{r}^*_v \cdot \chi^{\{v\}}) - R(\hat{\mathbf{r}}) \right] \leq \max_{\mathbf{r}: \sum \mathbf{r}_v \cdot c(v) \leq B} \left[\sum_{v \in supp^+(\mathbf{r})} [R(\hat{\mathbf{r}} \vee \mathbf{r}_v \cdot \chi^{\{v\}}) - R(\hat{\mathbf{r}}) \right] \tag{10}$$

Note that $supp^+(\mathbf{r}') = supp^+(\mathbf{r}^* - \hat{\mathbf{r}})$. The equation above is saying that the element-wise sum of marginal gain due to $\mathbf{r}'$ is less than or equal to the that of a vector $\mathbf{r}$ which satisfies the budget constraint and maximizes the element-wise sum of marginal gain. Now,

$$\max_{\mathbf{r}: \sum \mathbf{r}_v \cdot c(v) \leq B} \left[\sum_{v \in supp^+(\mathbf{r})} [R(\hat{\mathbf{r}} \vee \mathbf{r}_v \cdot \chi^{\{v\}}) - R(\hat{\mathbf{r}}) \right] = \sum_{i=1}^{k-1} \Delta(s_i) + \frac{B - \theta}{c(s_K) \cdot \sigma_{s_K}} \Delta(s_K) \tag{11}$$

The equation above holds because the element-wise sum of marginal gain can be optimized by simply picking the top-K nodes which maximize the marginal gain. Basically, the LHS is maximized by r^o such that $r^o_v = \sigma_v$. And the RHS is the value of objective in the LHS for r^o_v.

Combining (4), (5), (6), and (7), we get

$$R(\mathbf{r}^*) \leq R(\hat{\mathbf{r}}) + \sum_{i=1}^{K-1} \Delta s_i + \frac{B - \theta}{c(s_K)\sigma_{s_K}} \Delta_{s_K}$$

□
