# Supplementary figures and images for: Fast and near-optimal monitoring for healthcare acquired infection outbreaks
Source: PLoS Comput Biol. 2019 Sep 16;15(9):e1007284. doi: 10.1371/journal.pcbi.1007284 (PMC6762212; doi:10.1371/journal.pcbi.1007284)

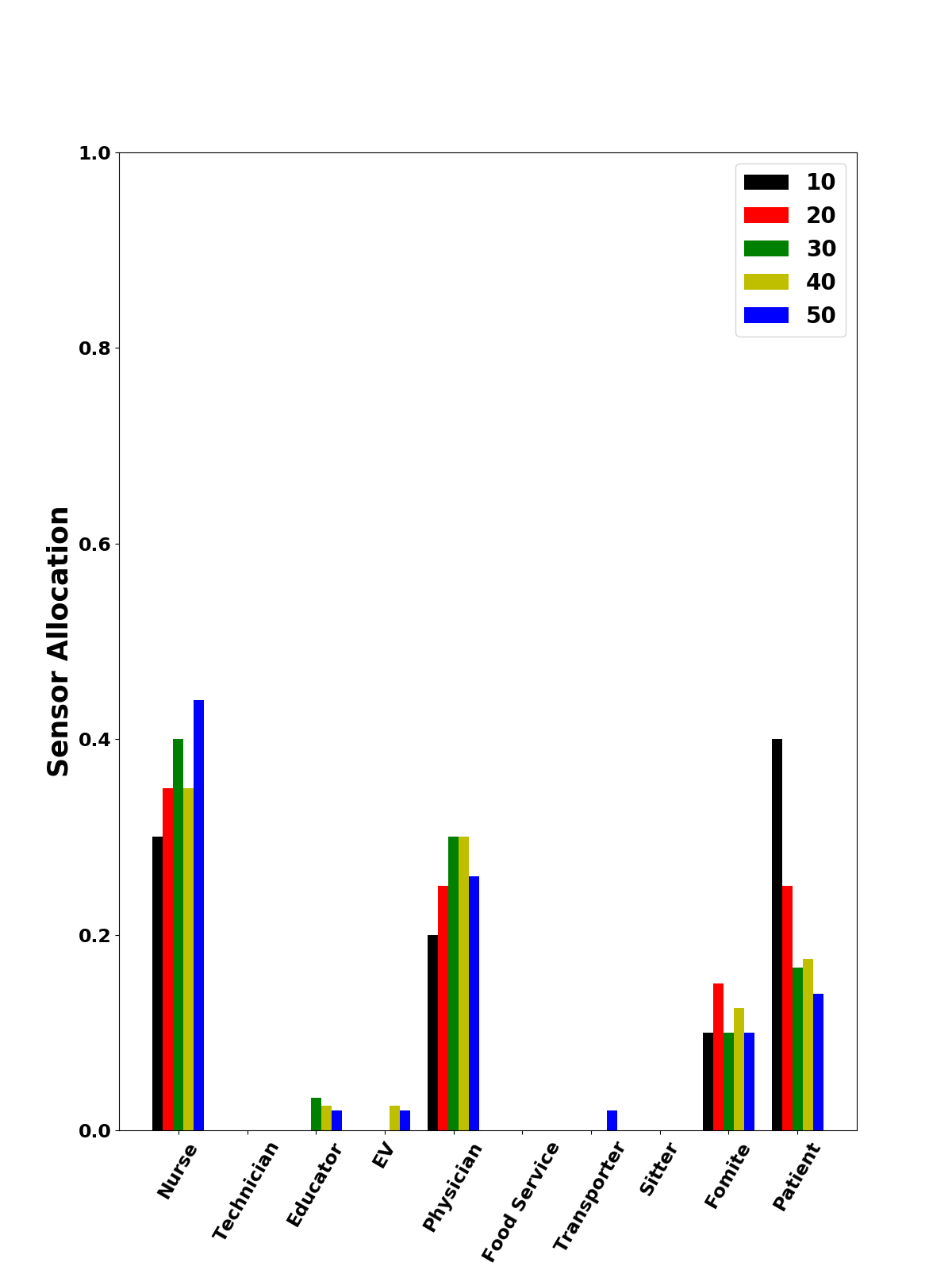

Supplement: S1 Fig — Note that Celf picks more patients and less nurses than HaiDetect for lower budgets, which explains its poor performance in the test set (Fig 6). (TIF) [file pcbi.1007284.s002.tif]

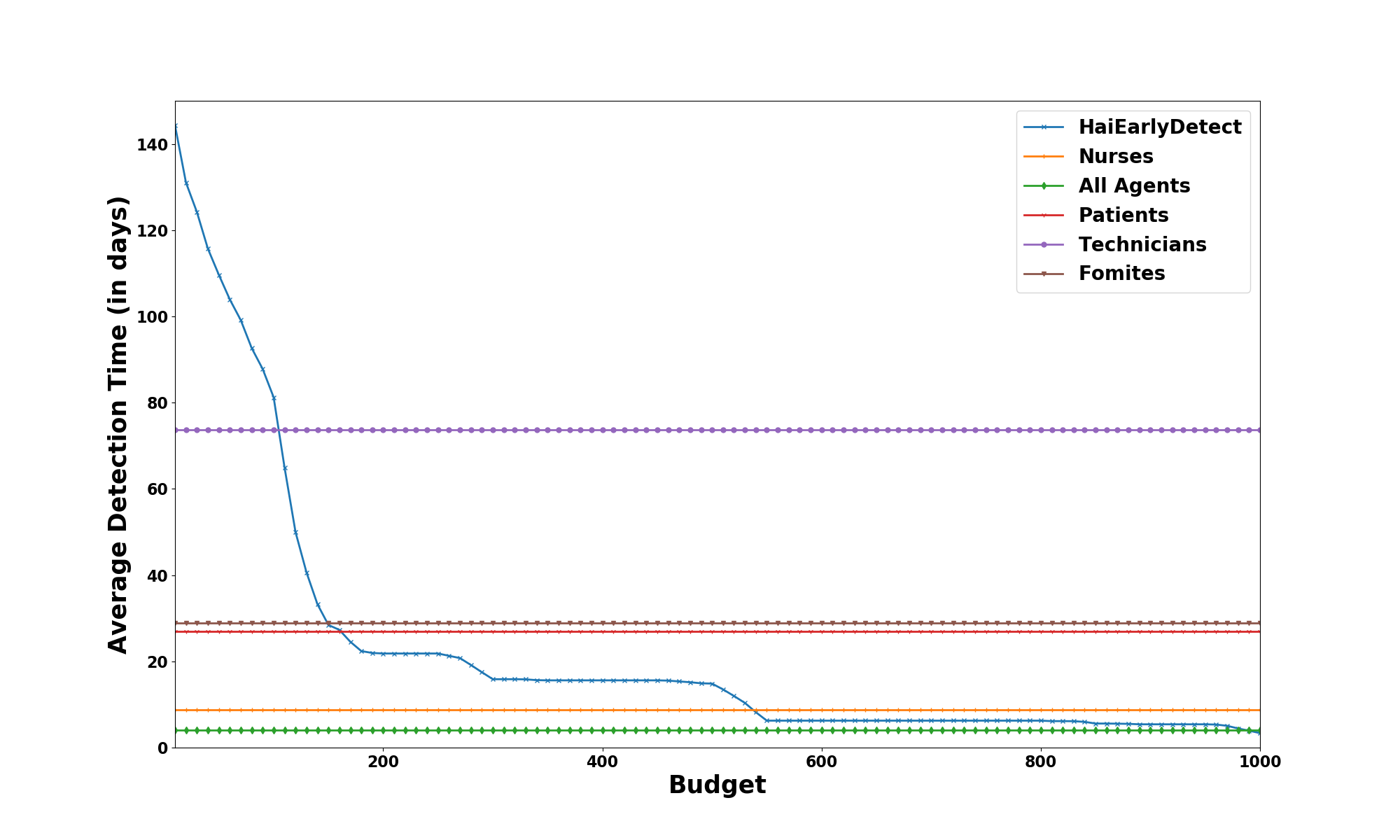

Supplement: S2 Fig — As expected, HaiEarlyDetect has even better detection time on training data. (TIF) [file pcbi.1007284.s003.tif]

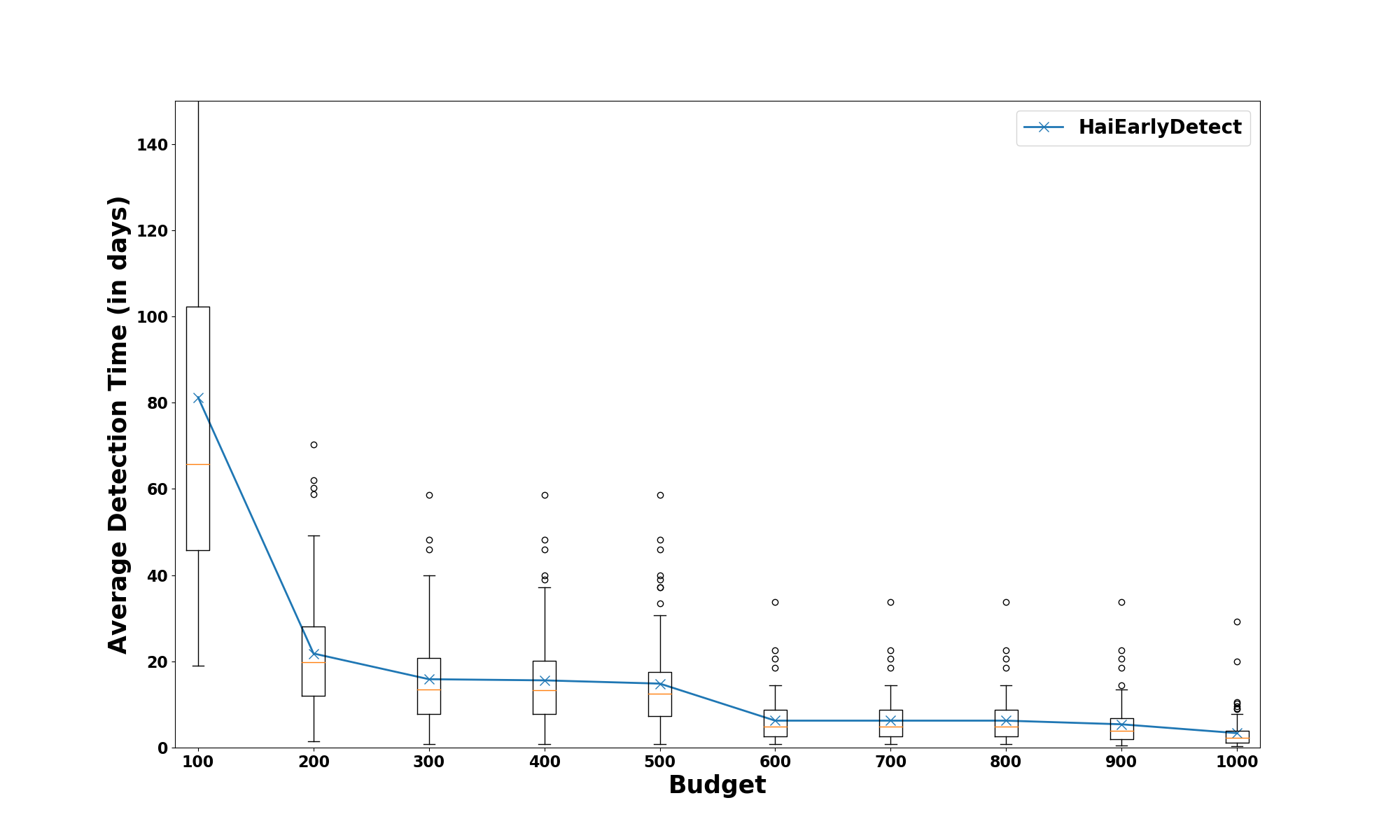

Supplement: S3 Fig — (TIF) [file pcbi.1007284.s004.tif]

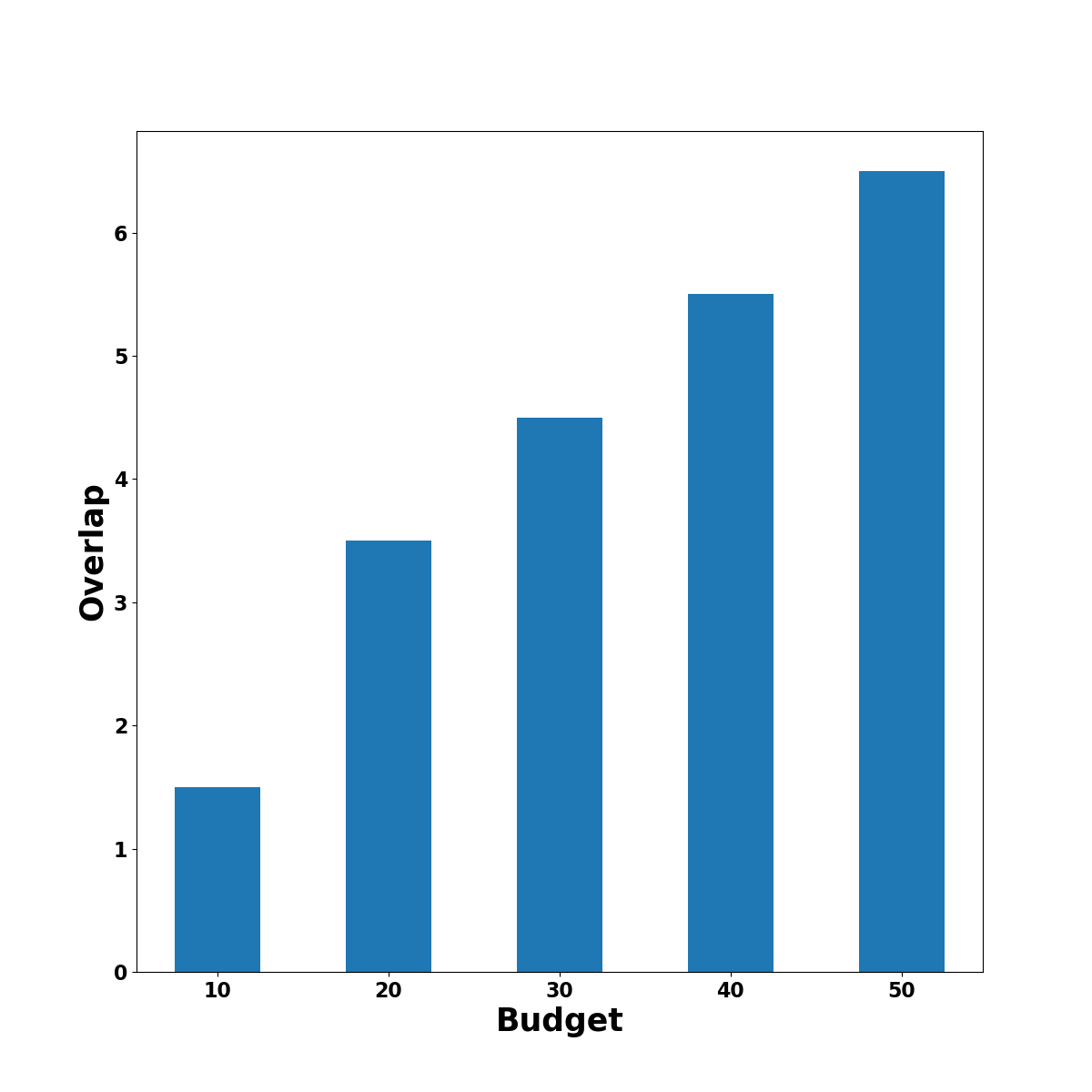

Supplement: S4 Fig — There is only roughly 10% overlap between Celf and HaiDetect, highlighting that HaiDetect selects different nodes and rates than Celf. (TIF) [file pcbi.1007284.s005.tif]
